# Nitrogen fixation and mucilage production on maize aerial roots is controlled by aerial root development and border cell functions

**DOI:** 10.3389/fpls.2022.977056

**Published:** 2022-10-06

**Authors:** Vânia Carla Silva Pankievicz, Pierre-Marc Delaux, Valentina Infante, Hayley H. Hirsch, Shanmugam Rajasekar, Pablo Zamora, Dhileepkumar Jayaraman, Claudia Irene Calderon, Alan Bennett, Jean-Michel Ané

**Affiliations:** ^1^Department of Bacteriology and Agronomy, University of Wisconsin-Madison, Madison, WI, United States; ^2^Department of Plant Sciences, University of California, Davis, Davis, CA, United States; ^3^Department of Horticulture, University of Wisconsin-Madison, Madison, WI, United States

**Keywords:** *Zea mays*, mucilage, brace roots, aerial roots, border cells, biological nitrogen fixation

## Abstract

Exploring natural diversity for biological nitrogen fixation in maize and its progenitors is a promising approach to reducing our dependence on synthetic fertilizer and enhancing the sustainability of our cropping systems. We have shown previously that maize accessions from the Sierra Mixe can support a nitrogen-fixing community in the mucilage produced by their abundant aerial roots and obtain a significant fraction of their nitrogen from the air through these associations. In this study, we demonstrate that mucilage production depends on root cap and border cells sensing water, as observed in underground roots. The diameter of aerial roots correlates with the volume of mucilage produced and the nitrogenase activity supported by each root. Young aerial roots produce more mucilage than older ones, probably due to their root cap’s integrity and their ability to produce border cells. Transcriptome analysis on aerial roots at two different growth stages before and after mucilage production confirmed the expression of genes involved in polysaccharide synthesis and degradation. Genes related to nitrogen uptake and assimilation were up-regulated upon water exposure. Altogether, our findings suggest that in addition to the number of nodes with aerial roots reported previously, the diameter of aerial roots and abundance of border cells, polysaccharide synthesis and degradation, and nitrogen uptake are critical factors to ensure efficient nitrogen fixation in maize aerial roots.

## Introduction

Roots are a primary connection between plants and their environment and, in particular, the soil microbiota. The layer in between the roots and the soils forms the rhizosphere, were most of the biogeochemical and bioactivity processes influence the host. These relationships influence plant nutrition and health. Roots produce and release exudates, mainly through their root tips, and to a lesser extent, at the root cracks such as the site of emergence of lateral roots (Rovira, 1969). The root exudates are composed of sugars, amino acids, organic acids, vitamins, and high molecular weight polymers that are released from root cracks such as the site of emergence of lateral roots and also from root tips, where they form a gelatinous substance referred to as mucilage or mucigel (Galloway et al., 2020). The root tip mucilage and microbial polysaccharides contribute to rhizosheath formation (Brown et al., 2017). The rhizosheath is a layer of soil around the root that adheres to the root upon excavation of the root system. It improves plant tolerance to drought stress, limits nutrient deficiency, and contributes to recruiting beneficial microbes (Brown et al., 2017). The underground root mucilage is primarily produced by specialized cells called border cells (Hawes and Pueppke, 1986). Upon water sensing or abrasion, border cells originating from the root cap meristem are released from the root cap, and mucilage is produced (Hawes et al., 1998). The fact that root border cells (RBC) produce mucilage has been described extensively for RBC from underground roots (Hawes et al., 1998; Kumar and Iyer-Pascuzzi, 2020). Mucilage is synthesized in various vesicles emerging from the trans Golgi network but the exact mechanisms of water sensing and mucilage release upon water sensing are still poorly understood (Wang et al., 2017). Despite their detachment from the root, border cells remain viable for several weeks in the soil and contribute to mucilage secretion (Driouich et al., 2019). Underground root border cells have numerous functions, including reducing the friction resistance to promote root growth, facilitating beneficial bacteria colonization, and counteracting physical damage due to drought stress and antimicrobial production and chemical toxicity (Curlango-Rivera et al., 2013; Yan et al., 2014). Even more surprisingly, border cells attract and ensnare potential pathogens with extracellular DNA and protein traps, respectively, to halt infection (Tran et al., 2016). Despite the wide range of functions for these cells, the genetic regulation of mucilage production is poorly understood. The main components of the root mucilage are polysaccharides, proteins, and border cells. Still, the composition differs between plant species, between accessions of the same species, and even across the root system of the same accession. For example, when comparing *Arabidopsis thaliana*, pea (*Pisum sativum*), rapeseed (*Brassica napus*), and maize (*Zea mays* ssp. mays), there were significantly more proteins identified in maize mucilage overall, and 85-94% of the proteins identified within the three dicots had close homologs within the maize mucilage proteome, suggesting a high degree of conservation between the monocot and dicot mucilage proteins. (Ma et al., 2010). Significant differences in border cell numbers were observed between cotton cultivars (Curlango-Rivera et al., 2013; Knox et al., 2020). These differences, potentially due to environmental conditions, may influence the resistance to pathogens (Hawes et al., 2000). Within the Chinese inbred maize accession, Xiaohuangbaogu, the number, and viability of border cells varied between underground and aerial roots (Yan et al., 2014). While in underground roots, the border cell number increased rapidly during root elongation, up to 4,000 border cells per root tip, and decreased sharply when the root stopped elongating, maintaining around 1,900 border cells; the average in aerial roots was 2,500 border cells during root elongation. Because of the experimental setup, the authors did not evaluate the aerial root during the entire plant development (Yan et al., 2014).

Along with this observed variation, aerial root mucilage composition within Sierra Mixe maize grown in the state of Oaxaca, Mexico, and in California, United States, were found to share near-identical polysaccharide structures. It is hypothesized that the shared polysaccharide influences microbial colonization within aerial root mucilage (Amicucci et al., 2019). In conjunction with influence over symbiosis, mucilage production and border cell characteristics have been found directly dependent on the plant’s specific response to environmental stimuli such as moisture, temperature, drought, and toxins (Yan et al., 2014).

Sierra Mixe Totontepec Villa de Morelos (TVM), hereafter called Sierra Mixe (SM) is a landrace of maize described by Van Deynze et al., 2018 because of its potential for nitrogen fixation on aerial root. Compared to commercial maize accessions, Sierra Mixe accessions are quite tall, 3- to 5- meters, with a growing season over nine months (Van Deynze et al., 2018). They produce 8 to 10 nodes with aerial roots instead of 1 to 3 nodes found in most maize accessions grown in the United States. Sierra Mixe maize provides a new example of the microbiota’s influence over mucilage composition. Among expected components such as sugars, nucleic acids, and amino acids, Sierra Mixe aerial root mucilage was found to contain several diazotrophic species, homologs for nitrogenase subunits, and nitrogenase activity (Van Deynze et al., 2018). We estimated that Sierra Mixe maize could fulfill 29%-82% of its nitrogen nutritional requirements with atmospheric nitrogen fixation (Van Deynze et al., 2018). Though it is unclear how diazotrophs are sustained, these nitrogen-fixing associations are present in the Sierra Mixe maize in higher numbers than commercial maize, which relies entirely on nitrogen-rich fertilizer. This agricultural practice selects against biological nitrogen fixation in soil (Bloch et al., 2020).

Due to the novel trait displayed within Sierra Mixe maize, other maize accessions were examined for similarities to understand the association between maize and nitrogen-fixing bacteria inside mucilage. We investigated how the mucilage is produced and how this process differs between maize accessions. We found a strong positive correlation between aerial root diameter, mucilage production, and total nitrogenase activity. Across the root development stages, younger roots produce more mucilage than older roots. Mucilage production occurs at the root tip, around the root cap. Aerial roots can be categorized into four stages based on the abundance of mucilage production and root cap integrity. RNA-seq was performed to study the dynamic response of aerial root during water-stimulated mucilage production in a Sierra Mixe accession due to observed variation in mucilage production between genotypes and development stages compared to the reference B73 maize genome. Differential gene expression in response to water treatment and developmental stages were found between the maize genotypes. Our results demonstrate nitrogen fixation and the abundance of border cells within the aerial root mucilage produced by maize accessions. We observed direct mucilage secretion from border cells detaching from the root cap and variation in border cell production and shape between developmental stages and genotypes. Altogether, these results provide insight into shared and varied developmental and gene expression characteristics between maize accessions. This study provides the molecular and cellular basis for further investigation of the nitrogen-fixation associations in maize aerial roots, which offers the potential for improving other cultivated maize varieties.

## Material and methods

### Maize accessions cultivation and accessions

**Figure 1:**
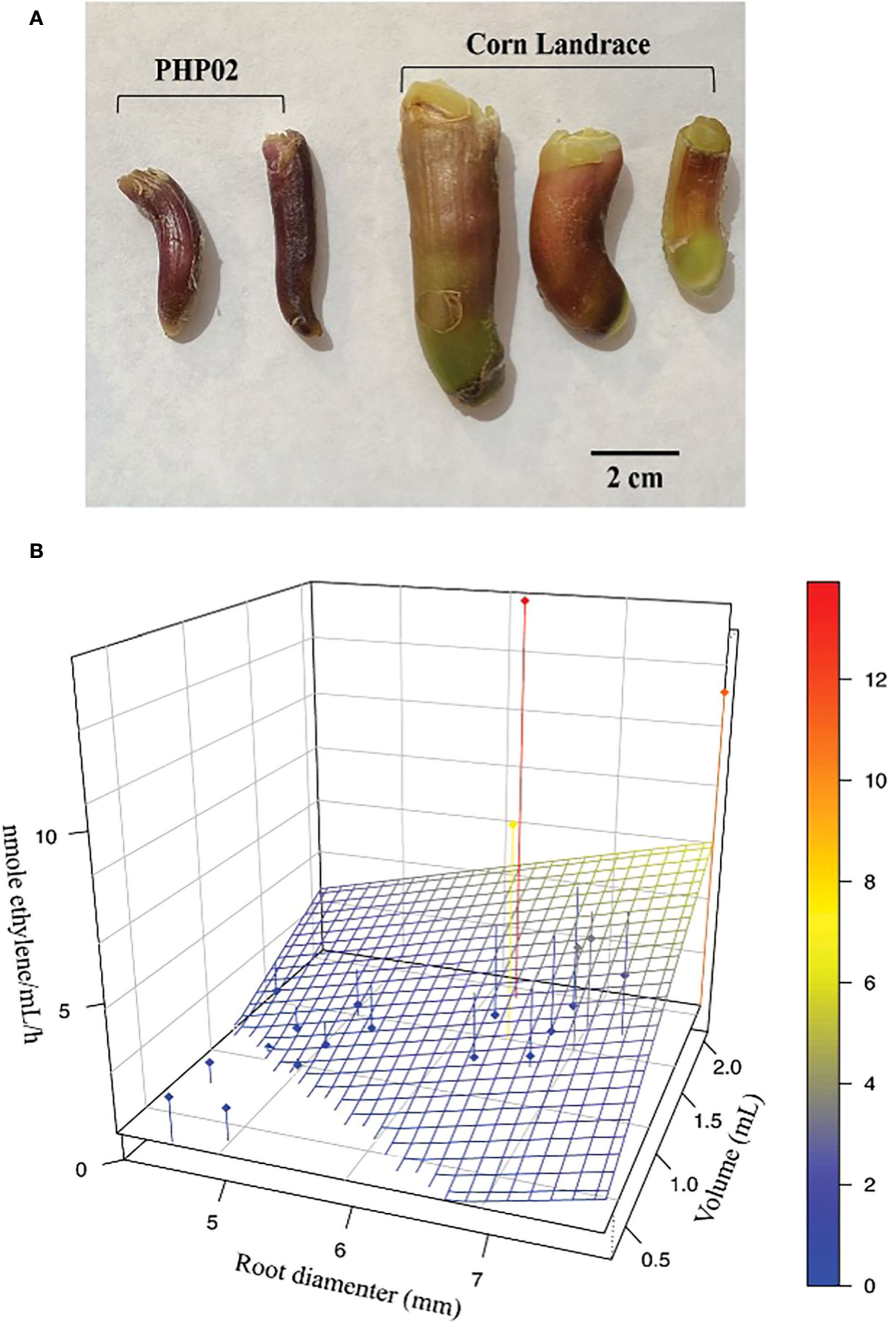
Aerial roots produced on different maize accessions secrete mucilage and support biological nitrogen fixation. **(A)** Aerial roots formed on the stem of maize accessions Ames 19897 that produce 1,5 mL of mucilage are longer and thicker than those produced in common maize variety PHP02 (25 µL). **(B)** There is a correlation between root diameter, volume of mucilage produced, and the ability to support nitrogen fixation. Aerial roots from 22 distinct maize accessions showed variable diameters. Those with larger diameters tended to secret more mucilage, showing higher nitrogenase activity (nmol of ethylene per hour per mL of mucilage). The Blue to red color scale indicates the nmole/ethylene/mL/h.

Sierra Mixe maize (*Zea mays*) accession Totontepec Villa de Morelos (TVM) was obtained in Oaxaca, Mexico, from an open-pollinated population. Biological materials of Sierra Mixe maize were accessed and utilized under an Access and Benefit Sharing Agreement between the Sierra Mixe community and BioN2, Inc., and permission from the Mexican government. An internationally recognized certificate of compliance under the Nagoya Protocol (ABSCH-IRCC-MX-207343-3) has been issued for such activities. For the RNA-seq experiment, Sierra Mixe and B73 were cultivated in a high ceiling room at the Biotron facility (University of Wisconsin, Madison, USA). Plants were watered twice a day for 2 minutes with half-strength of Hoagland’s solution. Because the Sierra Mixe seed was no longer available for border cell microscopy, we used the maize accession obtained from the Germplasm Resource Information Network (GRIN) collection, accession number Ames 19897 (**Figure 1A**). For border cell characterization, the plants were cultivated in Turface^®^ MVP at the Biotron facility under the controlled condition of 14/10h light/dark cycles, relative humidity 85%/90% day/night, and 25°C/17°C day/night. Plants were watered twice a day for 2 minutes, making up around 250 mL with half-strength of Hoagland’s solution. For phenotyping maize accessions in the 2019 season, seeds were obtained from the GRIN and CYMMIT (International Maize and Wheat Improvement Center) collections. The plants were grown in the West Madison Agriculture Research Station. Ten plants per accession were planted per row with two replicates in the field. The fertilization regime was done before planting, with 60 kg/ha nitrogen as urea, and the plants relied only on rain. *Z*. *mays* PHP02 and B73 seeds are conventional maize inbred genotypes and were generously obtained from Dr. Shawn Kaeppler and Dr. Natalia de Leon at the University of Wisconsin - Madison.

### Aerial root phenotyping and mucilage volume quantification

For aerial root phenotyping, 29 maize accessions from the GRIN and CYMMIT, in 3 independent field plots with 20 plants per genotype, were planted with three border rows (B73) between each genotype. Those plants were grown in the West Madison Agriculture Station at the University of Wisconsin-Madison during Summer 2014. The number of nodes with aerial roots and the total number of aerial roots per plant were recorded. Fourteen weeks post-planting, the aerial root diameter was measured with a manual caliper. The aerial root was in stage 2, and the diameter was measured at 1 cm from the base of the root. Additionally, the mucilage volume per root was manually collected, quantified, and used for the acetylene reduction assay. Mucilage root collection was challenging because of viscosity. The best method was using manual extraction by wearing sterile gloves, we pressed the root base from top to bottom, from which the mucilage was drawn straight into a graduated tube where the volume was measured. In the 2019 summer season, 20 plants per accession from the GRIN and CYMMIT collection were planted in the West Madison Agricultural Research Station-UW-Madison. Those plants were used for phenotyping the number of nodes with aerial root and aerial root diameter, which was measured with a manual caliper.

### Acetylene Reduction Assay (ARA)

After a rain event, one or two aerial roots from the several cultivated maize accessions were selected, and 2 mL of fresly collected mucilage was collected and then sealed in 14.5 mL vials that were tightly closed (Wheaton, Millville, USA). For ARA, no exogenous bacteria were added, and the procedures were the same as described in (Van Deynze et al., 2018). After enclosing mucilage in the vial, 850 μl of acetylene (Airgas) was injected into each vial. Controls without acetylene were performed in parallel. Ethylene quantification was made by injecting 1 ml of the air phase, sampled after 72 hours, on a gas chromatography (GC-2010 Shimadzu).

### RNA-seq samples preparation and RNA extraction

**Figure 2:**
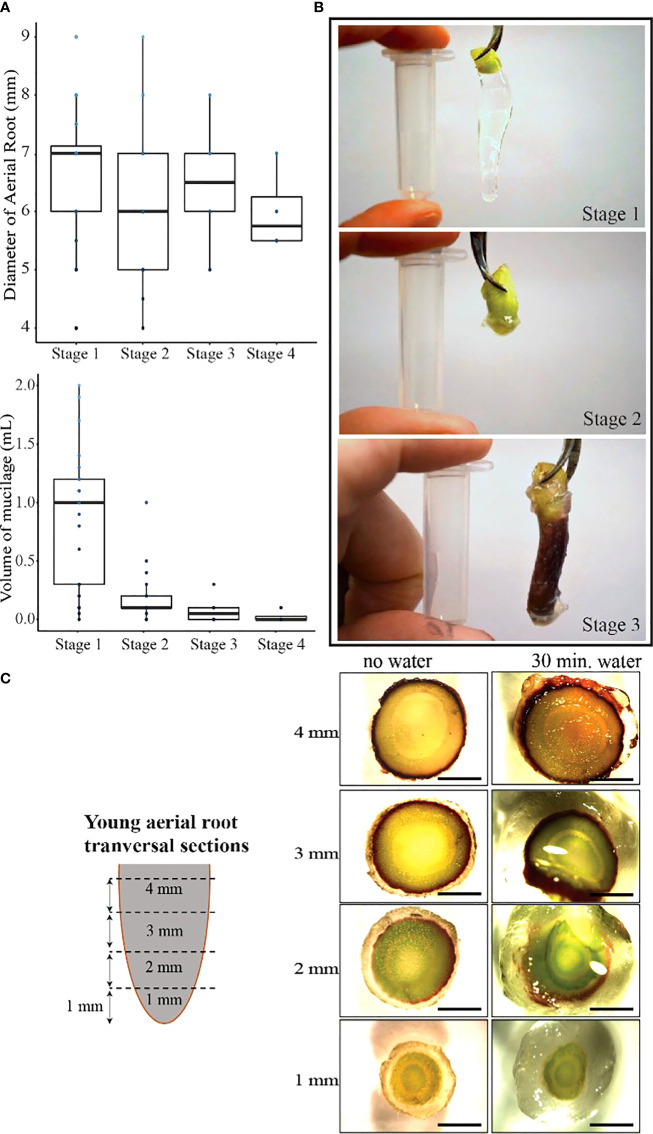
Younger aerial roots produced more mucilage and were categorized into three stages. **(A)** The diameter of aerial roots does not change during plant development, but the amount of mucilage produced does change. **(B)** Stage 1 roots are shorter and produce up to 1.5 mL of mucilage. In stage 2, roots still produce mucilage up to 0.5 mL, and in stage 3, the root cap starts to detach from the aerial root, and mucilage is no longer produced. Stage 4 has no visible root cap and does not produce mucilage. The 2 mL tube in the picture was 4 cm long. **(C)** The root tip of the aerial root produces more mucilage than the base of the root, and water is necessary to contact the aerial root to stimulate the secretion of mucilage.

**Figure 3:**
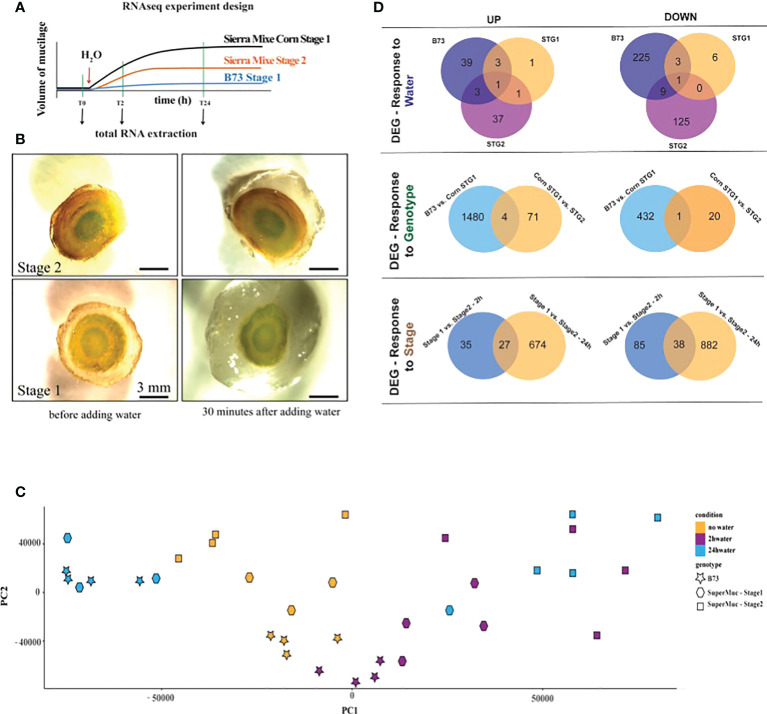
RNAseq analysis was performed to understand maize aerial root’s response during mucilage production. **(A)** RNAseq experiment schematic representation. B73 aerial roots produce tiny amounts of mucilage. Stage 1 roots from maize accession Sierra Mixe produces more mucilage than stage 2. **(B)** Transversal sections of aerial roots in stages 1 and 2 before contact with water and 30 minutes after contact with water. **(C)** Principal component (PC) analysis plot displaying all 35 samples along PC1 and PC2. **(D)** Differentially regulated genes (DEG) were selected to respond to water, genotype, and stage. The Venn diagram summarizes the overlap and unique UP or DOWN regulated genes in the various conditions; upper panel B73, Stage 1, and Stage 2 overlap referring to before and after adding water; middle panel, overlap between genes regulated in B73 and those regulated in Stage 2 and lower panel, the overlap of genes in Stage 1 and Stage 2 at 2 and 24 hours after exposure to water. p-value < 0.05 and log(10) fold-change > 1.0.

Sierra Mixe and B73 maize growing in a high ceiling room (14/10h light/dark cycles and 25/17° C and 85%/90% relative humidity) were subject to rain events (30 minutes of artificial rain) twice a week to trigger mucilage production. After fourteen weeks post planting, stage 1 Sierra Mixe aerial roots, stage 2 Sierra Mixe aerial roots, and stage 1 B73 aerial roots were collected before a rain event (control – T0) and then 2 (T2) and 24 hours (T24) after a rain event (**Figure 2**). After excision, the roots with its mucilage were individually snap-frozen in liquid nitrogen and then stored (**Figure 3A**) B73 aerial roots were similar to the Sierra Mixe ones but with a much smaller diameter and fewer mucilage produced. The total RNA present in secreted mucilage was extracted using PureLink^®^ RNA Mini Kit (Ambion) and treated with DNAseI (Ambion^®^) following the manufacturer’s instructions. A nanodrop spectrophotometer and Agilent Bioanalyzer’s electrophoresis on-chip were used to check RNA quantity and purity. Libraries for sequencing were prepared using the TruSeq RNA Library Prep Kit v2 (Illumina^®^) and sequenced in next-generation sequencing platform HiSeq 2500 system (Illumina^®^) using the TruSeq SBS Kit v3-HS (pair-end 100). Four independent samples were processed in parallel for each treatment type, resulting in 36 sequencing libraries. The raw sequence data are available in the Cyverse database and at NCBI GEO – accession number GSE168384.

### RNA-seq analysis

Sequencing libraries were uploaded to the discovery environment in the Cyverse platform. Raw data were analyzed for quality using FastQC (Andrews, 2015). For the pseudo sequence alignment, Kallisto (Kallisto-0.42.3) was used; under the Cyverse database (Bray et al., 2016), parameters were set to 100 bootstraps with four threads. Reads were mapped to *Zea_mays.B73_RefGen_v4.cdna*. Sleuth (version 0.30.0) was used to analyze the transcript abundance quantified by Kallisto and determine whether there were statistically significant differentially expressed genes (DEGs) between two groups using a Wald test (Pimentel et al., 2017). RStudio was used to run R-package sleuth and perform subsequent differential gene expression analysis. Differentially expressed genes were those with a false discovery rate of less than 0.05 (FDR *q* values), and the cut-off was set to a TPM (transcripts per million) expression value of 1. Because our dataset consisted of nine different conditions, we ran seven pairwise comparisons using the full model in Sleuth, and all replicates were shown in the PCA analysis (**Figure 3**). The output data tables, consisting of log2 fold change (*beta* value) for each gene and corresponding *q* values, are shown in **Supplementary Tables 4**–**10**. The gene set enrichment analysis was performed using the PANTHER webtool (Raudvere et al., 2019).

### Light and fluorescent microscopy of border cells and mucilage

To examine the border cells, *Z. mays* accession Ames 19897 was cultivated in the greenhouse as described above. The plants were subjected to mist for 4 hours every day to trigger mucilage production. Aerial roots displaying excessive (more than 1 mL) mucilage production were excised and used for microscopy analysis. Aerial roots between 3-4 cm long from stages 1, 2, and 3 and underground roots between 10-15 cm long were used; 3 replicates were evaluated in 2 independent experiments. The root tips were gently agitated on individual microscope slides containing 100 µL of ultrapure water, covered with a glass coverslip, and visualized using 100 µL India ink (Sheaffer – Skrip^®^), which does not penetrate the polysaccharide layer. The slides were examined under a Fisherbrand™ upright microscope with a 1080p HDMI color camera attached. Images were taken using the Sebaview software from Fisherbrand™, and pictures from each independent experiment were taken. Twelve images of border cells (six from each separate experiment) for Ames 19897 accession stage 1, 2, and 3 and underground roots were used to calculate the border cell size using the Fiji application (Schindelin et al., 2012). For the viability assay, the border cells were collected on a slide in a drop of water, stained with Fluo Acridine Orange and Propidium Iodide (AO/PI assay DeNovix^®^), and then covered with a glass coverslip to observe under an inverted fluorescence microscope (Leica DMi8).

## Results

### Mucilage production on aerial roots of maize accessions is dependent on the diameter and age of aerial roots

Underground roots and aerial roots of most maize accessions produce some mucilage but in much smaller quantities than the Sierra Mixe aerial roots (Van Deynze et al., 2018). Sierra Mixe aerial roots produce 1.5-2 mL of mucilage per root after rain. Aerial roots from Sierra Mixe accessions are also usually longer and thicker than those found on common maize varieties, PHP02 for example (**Figure 1A**). However, Sierra Mixe maize is not the only accession to produce large amounts of mucilage. In Mexico, other maize accessions from the Oaxaca region have many nodes with aerial roots and can produce large amounts of mucilage after rain. The total number of nodes with aerial roots, the diameter of these roots, and the amount of mucilage produced after the rain was evaluated in the field experiment in 2014 (**Supplementary Figures 1**–**4**). We also identified five lines from the GRIN collection, and 64 from the CIMMYT collection, based on geographical origin, with the potential to secrete large amounts of mucilage. Those lines were grown at the West Madison Agricultural Research Station in the 2019 season **(** **Supplementary Figure S5****)**. A total of 20 plants per genotype were planted and evaluated for the number of nodes with aerial roots. The plants with more than five nodes with aerial roots were investigated further, and the number of nodes and the diameter of aerial roots were recorded. Twenty-five accessions were identified with more than five nodes with aerial roots; however, the variation within the same accession is considerably high, which is expected due to the natural genetic variation within these accessions. The aerial root diameter ranged from 4 to 9 mm, and the total number of nodes ranges from 3 to 9 (**Supplementary Figure S6**).

The phenotypic variation observed is that depending on aerial root diameter, one accession will produce a given amount of mucilage **(** **Supplementary Figures S3**, **S4****)**. A positive correlation was observed between the root diameter and the volume of produced mucilage, thicker roots produced more mucilage (**Figure 1B**). Lines L14, L11, and SM (Sierra Mixe) produced 2.1, 1.7, and 1.7 mL of mucilage per aerial root and measured 8, 7, and 7 mm in diameter, respectively. The accessions with thinner aerial roots, L3, and the inbred LH82 and B73 produced 1.6, 0.6, and 0.9 mL, respectively, and measured 4 mm in diameter. Similarly, the nitrogenase activity was higher in the accessions L13, L14, and L15, where 7.4, 11.1, and 13.9 nanomoles of ethylene were produced per mL of mucilage per hour. In contrast, no ethylene production was observed in the inbred lines used as references. Ethylene production in the acetylene reduction assay is used as a proxy for nitrogenase activity. Given these results, we observe natural variation in accessions’ ability to sustain efficient nitrogen fixation, and the efficiency strongly correlates to the accession’s ability to produce and secrete mucilage.

The mucilage secretion depends on the perception of water on the aerial root tip. Like in underground roots, the root cap and border cells are involved in this process (Guinel and McCully, 1986). We observed that when aerial roots elongate, their root cap deteriorates. We observed a correlation between age, root and root cap quality, and aerial root’s ability to produce mucilage (**Figure 2B**). Young aerial roots produced up to 2.0 mL of mucilage, while old ones produced less than 0.5 mL. Thus, we categorized the Sierra Mixe aerial root into four stages of interest. Stage 1 roots are about 0.5 cm long and produce 0.5 mL to 2 mL of mucilage; stage 2 roots were 1-2 cm long and have up to 1 mL of mucilage, and the root cap starts to be visible. Stage 3 roots are 2-4 cm long, and they do not produce more than 0.5 mL. Stage 4 roots are 4-10 cm long and do not produce mucilage. After these stages roots are withered and no longer produce mucilage. The measured diameter did not decrease with successive growth from stages 1, 2, and 3 (**Figures 2A, B**), but stage 1 aerial roots produce more mucilage than roots at any other stage (**Figures 2A, B**).

To further understand the mucilage production dynamics, we made one-centimeter transversal sections in stage 1 aerial roots. We observed that mucilage volume was higher at the root tip than the root’s elongated section, and the root base, located where the root emerges from the stem, exhibited even less mucilage (**Figure 2C**). After adding water to these trans-sections, we observed mucilage production as soon as 30 minutes after contact with water. The root tip and its above trans-section had the highest amount of produced mucilage. Therefore, we hypothesize that the root cap and border cells are responsible for aerial root mucilage production, as described in underground roots, and aerial roots in earlier stages have more potential for mucilage production than older ones.

### Differential gene expression observed in response to water, genotype, and developmental stage

Observed differences in developmental stages led us to conduct an RNA-seq experiment to study further how water influences aerial root mucilage secretion. The experiment design was set to be a comparison between the maize accession Sierra Mixe (SM) and the well-characterized B73 reference genome at three time points after water treatment (0, 2, and 24 hours) and between the SM accession stage 1 and 2 of aerial root development. Four biological replicates were used for each condition (**Figures 3A, B**). In this study, our priority was to perform comparisons within genotypes and not between genotypes; therefore, we choose to align the reads on the B73 reference genome. Differentially expressed genes (DEGs) between treatments were determined and uncovered 2,977 genes whose expression patterns were best explained by one of the factors: water, genotype, and stage (**Figure 3D**). Genes expressed in Sierra Mixe had higher expression values and a low number of down-regulated genes, indicating a reliable response to the treatments applied: genotype, and water. The differential gene expression between stages 1 and 2 was less prominent. Because our dataset consisted of nine different conditions or groups, we ran seven pairwise comparisons using the full model in Sleuth, with all replicates described in the principal component analysis (PCA) plot (**Figure 3C**).

The DEGs were selected to respond to water, genotype, and stage. The Venn diagram summarizes the shared and unique genes up or down-regulated within each comparison (**Figure 3D**). To further understand these responses, we ran a gene-set enrichment analysis using the PANTHER classification system, applying Fisher’s exact test, and considering FDR < 0.05.

For the water factor (W) in the set of up-regulated genes, the oligosaccharide biosynthetic process was significantly enriched with 54.16-fold enrichment. In the downregulated genes group, the GO terms, including response to abscisic acid, were enriched 41.53-fold, and response to various stresses was increased more than 20-fold (**Supplementary Table 1**). There were 213 down-regulated genes in the mucilage of aerial roots of SM at stage 2. The categories related to rRNA binding, ribosome structural constituent, and ribosomal biogenesis in the biological process category were enriched more than 20-fold, indicating a considerable downregulation of protein biosynthesis and regulation.

For the genotype factor (G), 436 genes were up-regulated in B73, 71 were up-regulated in stage 2, and only one overlapped between the two conditions. For B73, the overrepresented categories were cytosol cellular component, GTPase regulation and activity, metabolic alcohol process, and small molecule catabolic process. For genes unique to stage 2, the only category that was overrepresented was catalytic activity. It is expected that fewer genes will respond only to genotypic differences once the same genomic reference was used to analyze the data. It would be ideal to use the specific sequences for each plant variety. Nevertheless, within the 1,484 genes down-regulated in B73, the overrepresented categories were Hydro-lyase activity with 4.76-fold and cellular amide metabolic process with 2.06-fold enrichment. Hydro-lyase is the catalysis of carbon-oxygen bond cleavage through water elimination, indicating that even though B73 possesses these genes, the SM accessions had those genes overexpressed in the tested conditions.

For the stage factor (S), we compared the same stages response in the time points 2 and 24 hours after water treatment (**Figure 3**). After 24 hours, the number of genes up and down-regulated had increased, indicating a more robust response due to prolonged contact with water. We found 60 and 699 genes up-regulated after 2 h and 24 hours, respectively. Only 25 genes were shared between time points, and GO category enrichment was not observed, indicating smaller differences between the stages. Within up-regulated genes after 24 h, the glutamate metabolic process was enriched 15.6-fold. Among these, genes controlling N-assimilation such as a glutamate synthase, a ferredoxin-dependent glutamate synthase, and a glutamate decarboxylase were found. The carboxylic acid and organic acid catabolic process categories had 5.88-fold, and the response to organic substances had 2.93-fold enrichment. Organic acids are essential compounds of root exudates. Among down-regulated genes, like what was observed for stage 1 in response to water, many categories related to the ribosome pathway were overrepresented. This result suggests that stage 2 cells might have a lower metabolic rate. After 24 h, the down-regulated genes for stage 2 had categories related to glycogen biosynthetic process (27.54-fold), hexokinase activity (14.6-fold), and chaperonin-containing T-complex (18.8-fold). Considering that decreased mucilage production characterizes stage 2, it is interesting to observe that the biological processes related to protein and carbohydrate metabolism and transport were found down-regulated, especially 24 h after water treatment.

### Genes related to mucilage production and degradation are regulated in the RNA-seq data set

**Figure 4:**
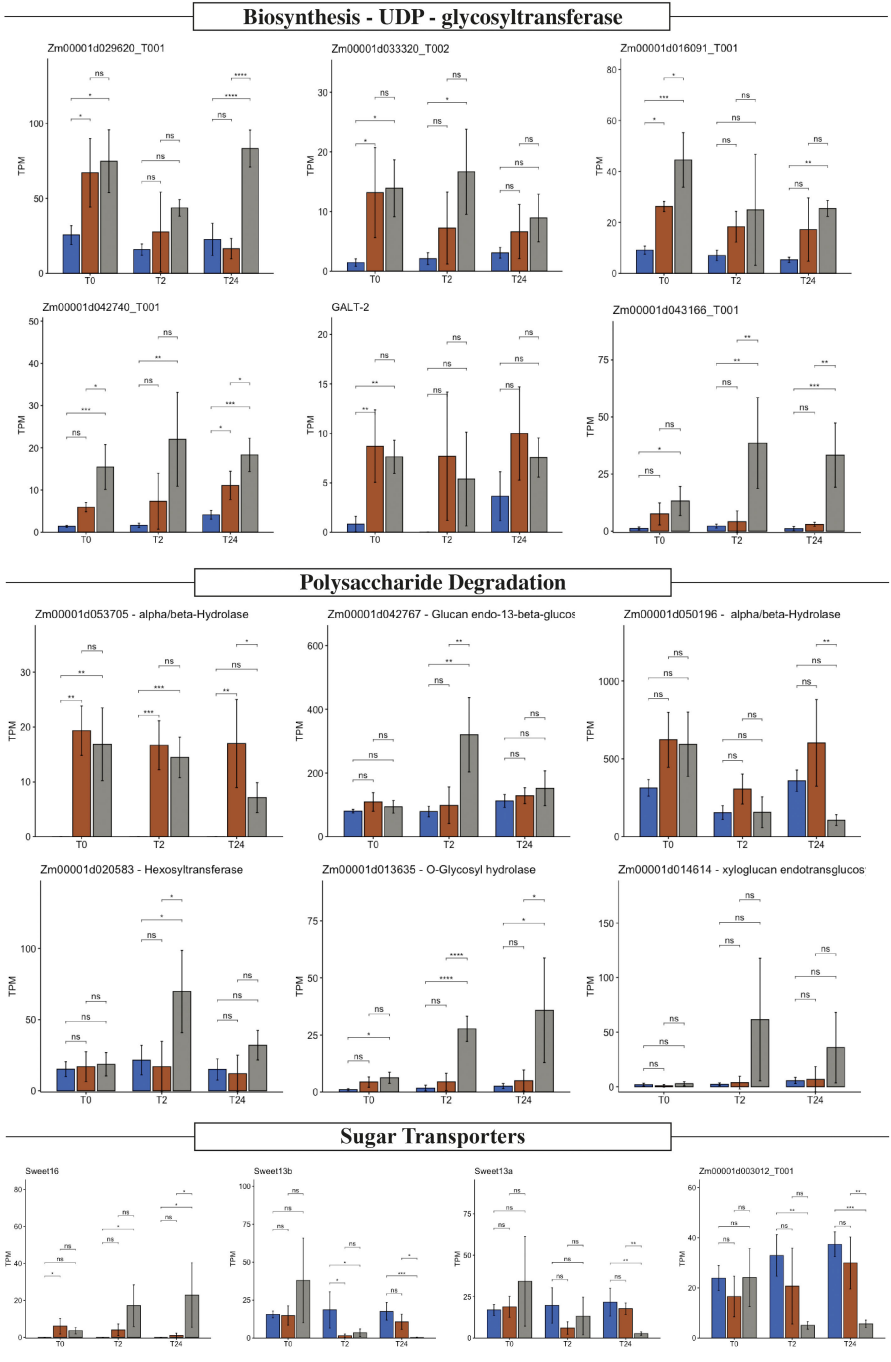
Polysaccharide biosynthesis, degradation, and sugar transport expression profile. Expression value in transcripts per million (TPM) of six selected glycosyltransferases, six hydrolases, and four sugar transporters in Z. mays B73 (blue), Sierra Mixe (SM) stage 1 (orange), and Sierra Mixe stage 2 (gray) at zero (T0), two (T2), and twenty-four (T24) hours after adding water. Analysis of variance (ANOVA) was performed on TPM values and significance difference represents *p < 0.05; **p<0.01, ***p<0.001, ****p<0.0001. ns stands for non-significative.

Recently, Amicucci et al. (2019) demonstrated that the Sierra Mixe maize mucilage composition is a single heterogeneous polysaccharide composed of a fucosylated and xylosylated galactose backbone with arabinan and mannoglucuronan branches. We proposed that this unique polysaccharide structure may select the diazotrophic community (Bennett et al., 2020). Transcripts related to polysaccharide biosynthesis have lower expression in B73 samples (**Supplementary Table S1**). Stage 1 and stage 2 samples also clustered separately, suggesting temporal regulation over specific genes or pathways. Four UDP- glycosyltransferases were highly expressed in stage 2: *Zm00001d043166*, *Zm00001d033320*, *Zm00001d016091* and *Zm00001d042740* (**Figure 4**). One specific UDP-glycosyltransferase, *Zm00001d029620*, was expressed in all conditions. This enzyme catalyzes glucose transfer from UDP-glucose to flavanol. This reaction is one of the last steps in anthocyanin pigment biosynthesis. We often observed a red pigment in the aerial roots of SM accessions, which may deserve further investigation (**Supplementary Figure S5A**).

UDP-glycosyltransferase gene expression was variable among all the evaluated conditions; this could be due to an imperfect mapping to the reads on the B73 reference (**Supplementary Table S3**) or phenotypic variations. Thus, it merits further investigation due to its relation to the phenotypic observations, such as aerial root quantity and diameter. Because this character manifests as an observable phenotype, it could serve as a visual field marker to select lines of interest. Other transferases related to biosynthesis varied in B73 and SM. These same transferases were highly expressed in the SM samples: *Zm00001d009539* (beta-14-xylosyltransferase), *Zm00001d011792* (UDP-D-apiose/UDP-D-xylose synthase 2), and *Zm00001d041504* (Hydroxyproline O-galactosyltransferase GALT2). UDP-D-apiose/UDP-D-xylose synthase enzyme forms UDP-d-xylose *via* UDP-d-glucuronate decarboxylation and has therefore been named UDP-d-apiose/UDP-d-xylose synthase. D-Apiose is a plant-specific, branched-chain monosaccharide found in rhamnogalacturonan II (Duff, 1965). GALT2 is involved in arabinogalactan glycosylation (Basu et al., 2015). Arabinogalactan proteins (known as AGPs) are proposed to play essential roles in various plant growth and development processes, including cell expansion, cell division, reproductive development, somatic embryogenesis, xylem differentiation, abiotic stress responses, and hormone signaling pathways (Seifert and Roberts, 2007; Ellis et al., 2010).

Transcripts related to polysaccharide degradation had a prominent regulation in SM samples in different stages (**Figure 4**). *Zm00001d053705* annotated as coding for an alpha/beta-hydrolases superfamily protein, is expressed only in the SM samples, while *Zm00001d042767* (Glucan endo-13-beta-glucosidase) and *Zm00001d050196* (alpha/beta-Hydrolases superfamily protein) were expressed in all samples. Four hydrolases were highly expressed only in SM stage 2: *Zm00001d020583* (Hexosyltransferase), *Zm00001d031727* (Sorbitol dehydrogenase), *Zm00001d013635* (O-Glycosyl hydrolases family 17 protein), and *Zm00001d014614* (Probable xyloglucan endotransglucosylase/hydrolase protein 21).

Transcripts related to sugar transport vary their regulation in response to water in the SM samples. These transcripts’ appearance suggested specific regulation over sugar metabolism during mucilage production. Three genes related to the general sugar transporters, *Zm00001d003012* and *Zm00001d023673 (*SWEET13b*) and Zm00001d023677* (SWEET13a), were expressed in B73 samples but not regulated by water addition. At the same time, *Zm00001d003012* and SWEET13b expression decreased in stage 2 samples after 24h, when there is no observable mucilage production anymore. The sugar transporter related gene *Zm00001d029098* (SWEET 16*)* was repressed in B73. The bidirectional sugar transporter SWEET 16 facilitates the entry or export of sugars across the plant plasma membrane or the endoplasmic reticulum membrane (Eom et al., 2015).

### Nitrogen transporters and metabolism-related genes were regulated in Sierra Mixe aerial roots after the addition of water

**Figure 5:**
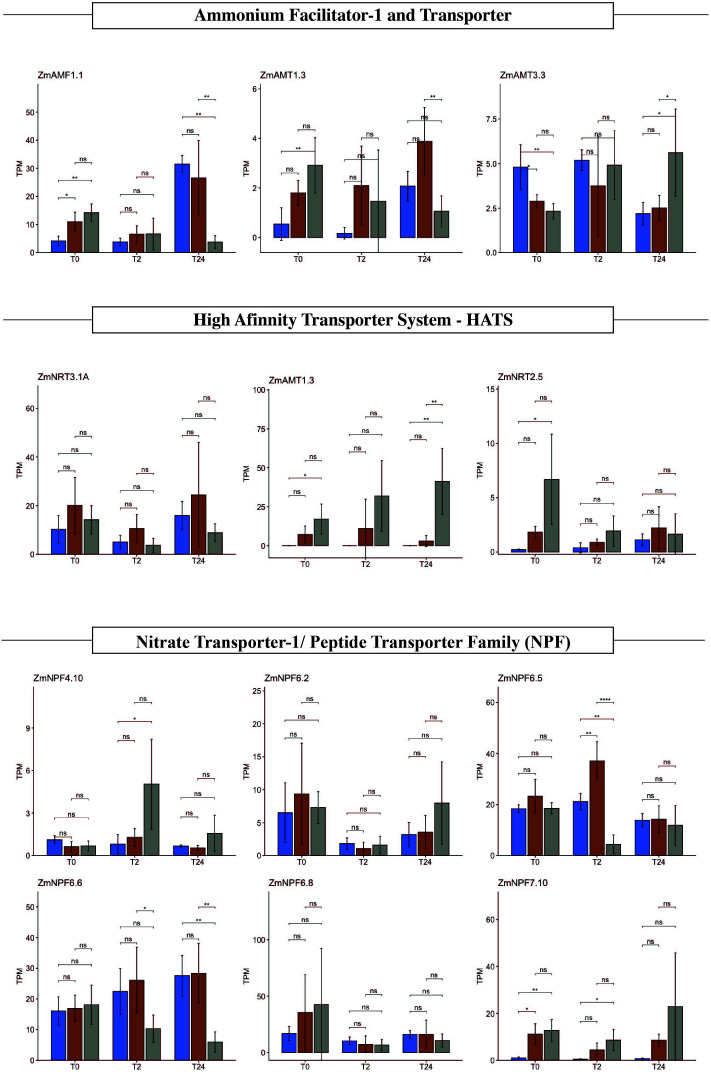
Nitrate, ammonium, and other nitrogen transporters expression profile. Expression value in transcripts per million (TPM) of two selected ammonium transporters (AMT), two ammonium facilitator 1 (AMF-1), nine nitrate transporters from nitrate and peptide transporter family (NPF), and high-affinity system transporters family (HATS) in Z. mays B73 (blue), Sierra Mixe (SM) stage 1 (orange), and Sierra Mixe stage 2 (gray) at zero (T0), two (T2), and twenty-four (T24) hours after adding water. Analysis of variance (ANOVA) was performed on TPM values, and significance difference represents *p < 0.05; **p<0.01; ****p<0.0001. ns stands for non-significative.

The expression of genes involved in nitrogen metabolism corroborates previous findings on the contribution of biological nitrogen fixation on aerial root mucilage (**Figure 5**). However, the uptake of nitrate or ammonium involves complex physiological mechanisms. For instance, when soil nitrogen concentrations are low (<250µM), a high-affinity transport system is activated (HATS). This system is under the control of NRT2 (Nitrate transporters) and AMT1 (Ammonium transporters) (Dechorgnat et al., 2011). On the other hand, when nitrogen concentrations are high (>250µM), a low-affinity transport system (LATS) becomes active. For nitrate, this system involves the NPF (NRT1/PTR Family) gene family (Dechorgnat et al., 2011). Although no transporter for ammonium has been described yet, the recently described AMF (ammonium facilitator 1) protein in soybean and yeast are promising candidates (Chiasson et al., 2014). Understanding all these pathways is not trivial in the aerial root – mucilage environment because of the dynamics of the wet/dry cycles and stages that aerial roots go through, likely changing the nitrogen supply. Our study aims to describe in general how the expression of these transporters varies across stages and in response to water treatment. Ammonium facilitators (AMF) and ammonium transporters were differently regulated in B73 and the SM aerial roots. After 24 h in contact with water, B73 and stage 1 SM samples had a 2.0-fold increase in *ZmAMF1*.1 and a 1.0-fold decrease in stage 2. The gene *ZmAMF1*.2, on the other hand, were regulated in all conditions but no differently expressed over time. Ammonium transporter AMT1.3 was found expressed in the aerial roots of SM samples, independently of the stage. While *ZmAMT3*.3 was expressed at all time-points and all conditions. Dechorgnat et al. (2019), working on maize, and Koegel et al. (2013), working on sorghum, have demonstrated that this ammonium transporter has a broad expression pattern in many plant organs, which is in line with our finding.

The high-affinity transporters *ZmNRT3*.1A was expressed in the B73 and SM samples. *ZmNRT3*.1B was expressed only in SM samples and up-regulated, especially during stage 2 after 24 hours. This corroborates the previous finding where no expression was observed for *ZmNRT3*.1B using B73 as the reference genome (Dechorgnat et al., 2019). It may give new ideas about its function since it was highly expressed only in the accession aerial roots and up-regulated in stage 2 compared to stage 1. Moreover, the *ZmNRT2*.5, which has been found induced after nitrogen starvation in underground roots (Dechorgnat et al., 2019), was up-regulated in SM samples compared to B73 at 0 and 2h after contact with water, suggesting that SM might be responding to the nitrogen dynamics on the aerial roots differently to what occurs in the underground roots.

Other nitrate transporters of the NPF family were up-regulated at one or more time-points in the SM samples than the B73 ones, including *ZmNPF4*.10, *ZmNFP7*.10, and *ZmNPF7*.12 (**Figure 5**). On the other hand, *ZmNPF6*.2, *ZmNPF6*.5, *ZmNPF6*.6, and ZmNPF6.8 were, in general, similarly regulated in SM and B73 at each time point. Some exceptions were: *NPF6*.5 stage 2, 2 h; *NPF6*.6, stage 2, 2 h, and 24 h. The stage 2 condition of SM samples is responsible for significant aerial root dynamics changes. This was already observed with the PCA analysis and the Venn diagrams, where all the DEGs are separated, especially after water addition. Under these conditions, the nitrogen metabolism needs further investigation to describe factual pathways for aerial roots’ nitrogen uptake.

Other sources of nitrogen to the plant can be peptides and amino acids (**Supplementary Table S1**). One probable peptide transporter *Zm00001d052435* was up-regulated in SM stage 2. On the contrary, two peptide transporters were expressed only in the B73 samples; *Zm00001d040947* and *Zm00001d023342*. The amino acid transporter antl-2 (*Zm00001d017557*) was overexpressed in stage 2, at 2 h and 24 h. Regarding the nitrogen assimilation pathway, the glutamate dehydrogenase 2 (*Zm00001d016419*) was expressed only in the SM samples. This enzyme is responsible for incorporating ammonium into 2-oxoglutarate to form glutamate, an essential step in the nitrogen assimilation pathway. More than that, the glutamine synthetase, gln4 (*Zm00001d017958*), showed an exciting regulation, with all described transcripts expressed; however, the isoform T006 had a higher expression in stage 2 at all time points and 24 h in B73 and stage1. This finding suggests a temporal regulation in stage 2 and probably abiotic effect from water in the other genotype. Related to nitrate uptake, the mha4 - proton-exporting ATPase4 (*Zm00001d026490*) was expressed, especially the isoform T022. Gene expression related to nitrogen assimilation pathways, and others related to nitrogenous compound uptake, suggests that aerial root mucilage creates an environment that may be of importance for nitrogen uptake by the plant.

### Mucilage is enriched in border cells, and border cell from different genotypes have different characteristics

As reported for the mucilage from underground roots (Hawes et al., 1998), we found that aerial roots’ mucilage is rich in border cells. To our knowledge, border cell development from aerial roots was only described by Yan et al., 2014 in the Xiaohuangbaogu inbred line. In contrast to what these authors reported, our maize accessions showed a much higher number of border cells in the aerial roots than what is described for underground root in B73. In contrast to the Xiaohuangbaogu aerial roots, which had about 2,500 border cells per mL of mucilage, the accessions we studied had about 25,000 border cells per mL (**Supplementary Figure S7**), ten times more than the Xiaohuangbaogu inbred. Compared to what is described by Hawes et al., 1998 for maize underground root, the accessions studied here possess about ten times the amount of border cells (Hawes et al., 1998). Iijima et al., 2000 described that maize root cap cells quantity increases from 1,930 to 3,220 cells per day per primary root as a result of soil compaction. In Yan et al., 2014, the Xiaohuangbaogu inbreed underground root had about 4,000 border cells in a 3.0 cm long primary root. In the well-described cotton cultivars, the border cell number per root tip was 27,921, a level not previously reported for any plant under the tested conditions. This is similar to what we observe in maize aerial-roots. Other studies revealed that underground roots could produce differing amounts of border cells under laboratory conditions, ranging from 0 to 10,000 per root among different species (Clowes, 1976; Hawes et al., 1998; Curlango-Rivera et al., 2013). However, the species-specific border cell quantity produced daily is conserved at the family level; if one species released several thousand cells, other species in that family generally did as well (Hawes and Pueppke, 1986; Brigham et al., 1995). This corroborates our finding that multiple accessions showed much higher numbers of border cells (**Supplementary Figure S7**).

**Figure 6:**
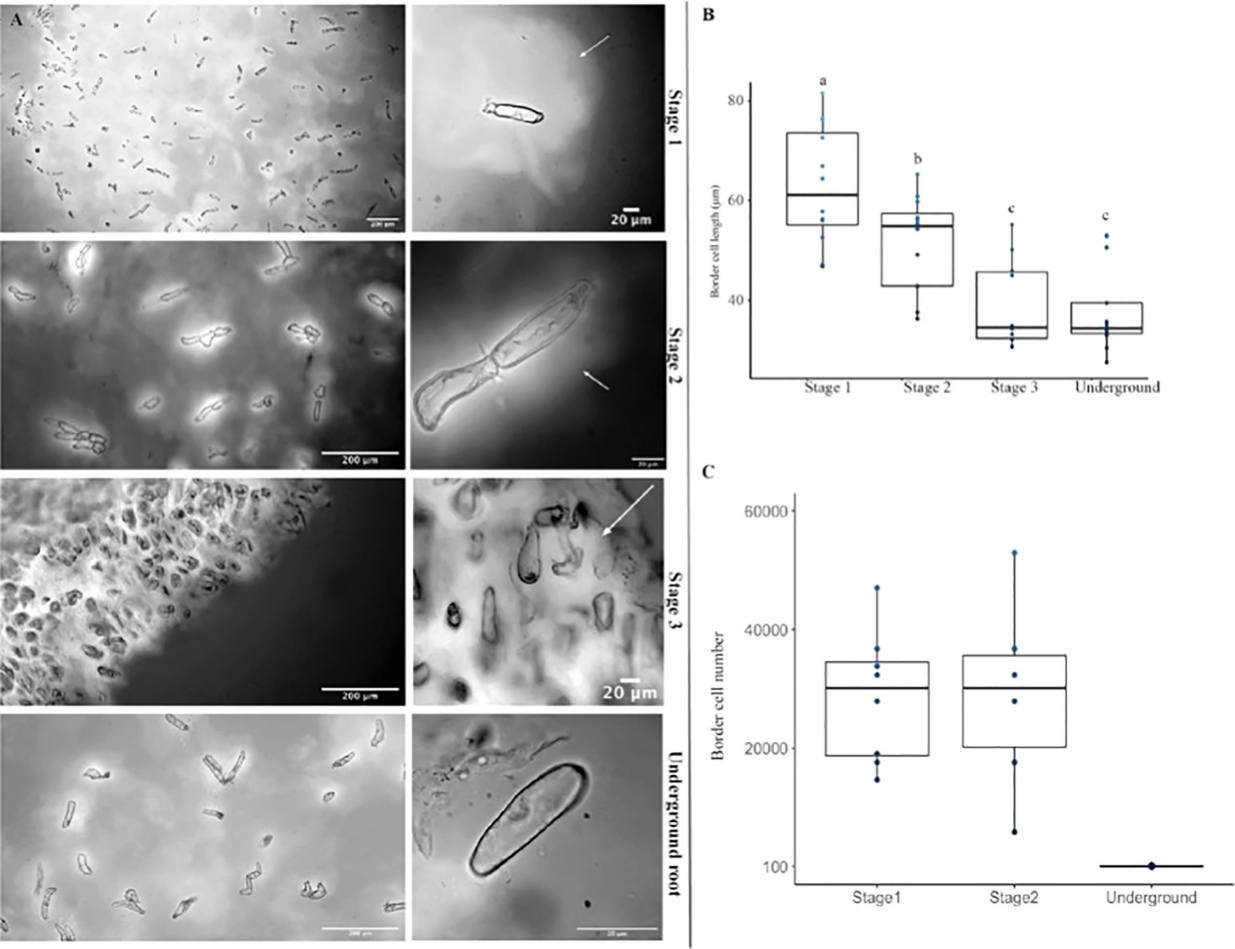
The mucilage produced in the aerial root surface is enriched in border cells, and the border cell secretes mucilage. **(A)** The left panel shows a general view of border cells embedded in its mucilage in stages 1, 2, and 3, and the underground root system. The right panels are magnified images of a light microscope representing the shape of the Ames 19897 accession border cells (GRIN) from aerial roots and underground roots. **(B)** Border cell lengths from aerial root stages 1 and 2 are larger than stage 3 and underground border cells. **(C)** Stages 1 and 2 have more border cells than stage 3. Error bars represent the standard deviation between biological replicates counted in two independent experiments. Analysis of variance (ANOVA) was performed on border cell length, and number values, and significance difference represents “a” p < 0.05; “b” p<0.01, “c” p<0,001.

We confirmed experimentally that the border cells from the aerial root mucilage are responsible for mucilage production. India ink stained revealed a white, unstained halo around the border cells, indicating the secretion of polysaccharides. **Figure 6A** shows border cells embedded in its mucilage in stages 1, 2, and 3 of aerial root development, as well as in the underground root system. Stage 1 and 2 show more prominent mucilage secretion from the border cells, while stage 3 releases a smaller number of detached cells in the mucilage (**Figure 6A**). The images exemplify the accessions border cell’s shape, of aerial roots and underground roots. The shapes of border cells derived from different roots were the same; however, border cells from aerial root stages 1 and 2 were larger than stage 3 and underground border cells (**Figure 6B**). Our data indicate that aerial root mucilage contains an abundant release of border cells that secretes mucilage. This mucilage is the primary environment where nitrogen fixation occurs in SM maize and other accessions. Aerial roots have been shown to develop under stress conditions, such as nutrient deficiency, and border cell number responds to environmental conditions (Steffens and Rasmussen, 2016). SM maize is often grown under low nitrogen, humid and rainy environments of Oaxaca. Detailed studies considering environmental changes will better describe the border cell dynamics and influence in mucilage secretion.

## Discussion

### Mucilage production on aerial roots is a trait present in many maize accessions

Cereals are the most produced commodities globally and are essential crops for food, feed, forage, fiber, and biofuel production. Unfortunately, they require significant external nitrogen applications to achieve high yields. The pressure on farmers to increase yields often leads to excessive fertilizer application (Pankievicz et al., 2019). The consequences of excessive synthetic fertilizer application and successive nitrogen loss are substantial. Farmers have a high monetary cost related to loss of fertilizer, use of equipment, and labor. And, in terms of global environmental degradation, there can be pollution of aquifers and coastal surface waters, an increase in greenhouse gas emissions and a decrease in food security. The research presented in this manuscript focuses on understanding the natural diversity in mucilage production across maize accessions (*Zea mays)*. Maize from the Sierra Mixe (SM) was previously identified as an outstanding system for biological nitrogen fixation (Van Deynze et al., 2018). These maize accessions show unusual and interesting phenotypic characteristics; indeed, however, at least twenty accessions from the GRIN and CIMMYT collection selected from other regions in Oaxaca also presented similar traits indicating that the trait is not unique to SM maize (**Supplementary Figures S5**, **S6**). Compared to commercial maize accessions, SM accessions are quite tall, 3- to 5- meters, with a growing season over nine months (Van Deynze et al., 2018). They produce 8 to 10 nodes with aerial roots instead of 1 to 3 nodes found in most maize accessions grown in the United States (**Supplementary Figures S1**, **S2**). The reason for this continuous aerial root production is that these accessions keep producing aerial roots at the adult vegetative stage, whereas regular maize ceases aerial root production in the juvenile stage (Van Deynze et al., 2018). The aerial roots are responsible for producing a copious amount of mucilage, and one aerial root can produce up to 2 mL of mucilage per root after rain (**Figure 2**). Using the acetylene reduction assay, we demonstrated that the nitrogenase enzyme complex is active in the mucilage and correlates with aerial root diameter and the amount of mucilage produced in various accessions (**Figure 1**).

### Younger aerial roots produce more mucilage than older ones, and border cells play an essential role in this process

To better understand where and which cells produce the mucilage, we performed longitudinal sections of the base of lateral roots to check for the presence of a specific gland as observed on leaves of the well-characterized *Gunnera* plants that associate with *Nostoc* diazotrophs, but we found no evidence of such glands in monocots (data not shown). We then made trans-sections of the aerial roots and even added water to these sections and found that maximal mucilage production was localized to the tip of aerial roots (**Figures 2B, C**). The root tips, including aerial roots, are covered by the root cap. When the root cap is in contact with water, border cells detach from the root cap. Gel production localization suggested that the root cap and border cells separating from it could be the source of mucilage production. Observations made on young aerial roots confirmed this hypothesis (**Figure 2B**), where the root cap is perfectly intact in Stage 1, starts to degrade in Stage 2 and quite degraded in Stage 3, and absent in Stage 4.

**Figure 7:**
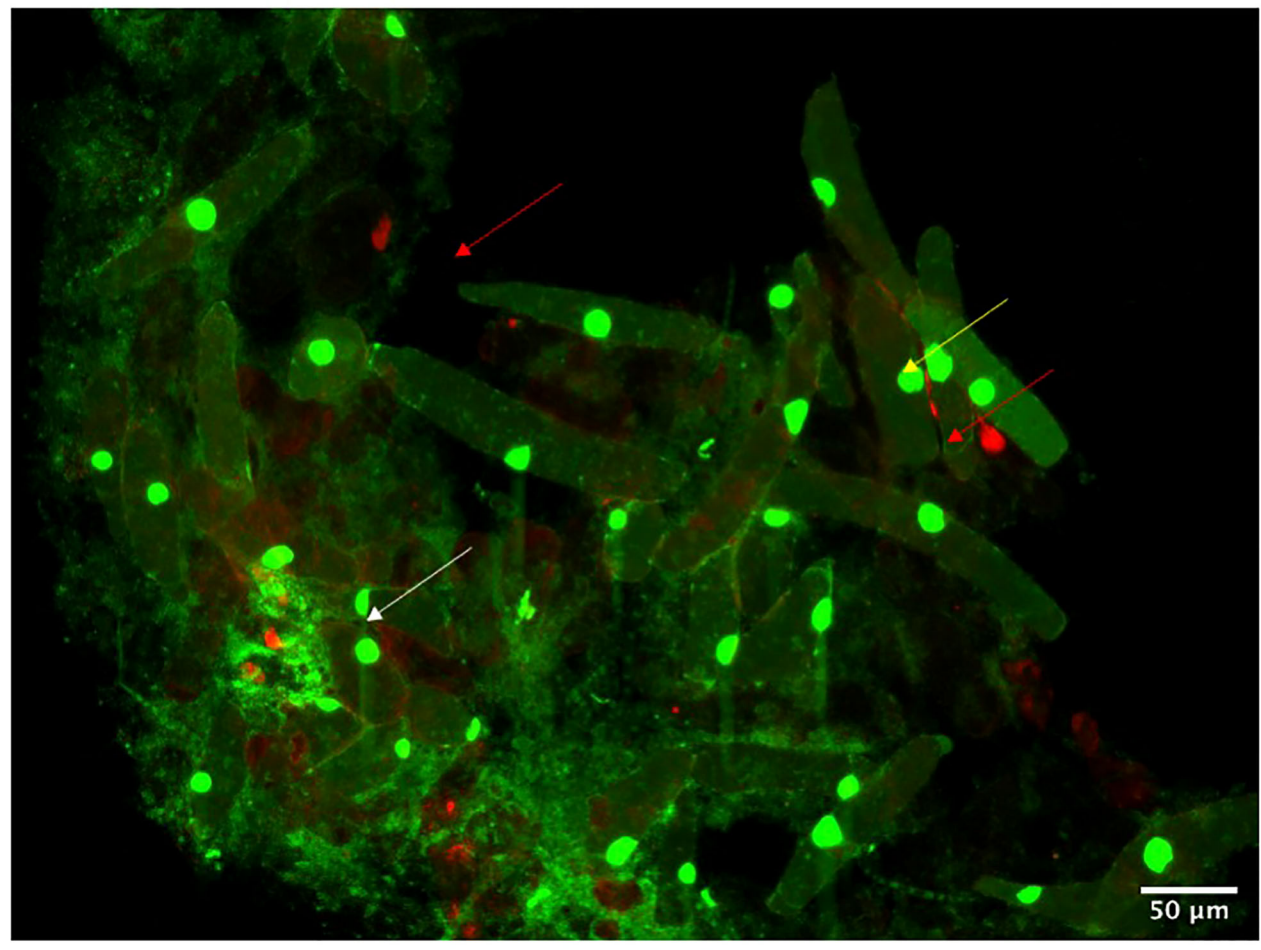
Root Border Cells from maize detach from the aerial root cap and remain in the mucilage environment. Fluorescent microscopy of border cells stained with propidium iodide marks dead cells (red arrows), and acridine orange marks viable cells (yellow arrows). White arrows indicate bacterial colonies trapped or attached to the border cells.

Furthermore, light microscopy analysis revealed a polysaccharide halo around the border cells demonstrating active mucilage production (**Figure 6**). Likewise, staining with India Ink showed that most of these border cells remain in the mucilage (**Figure 6A**) and are covered in a polysaccharides layer. This staining pattern is consistent with the mucilage composition described as a polysaccharide composed of a poly-galactose backbone grafted with side chains of poly-arabinose and fucose residues (Amicucci et al., 2019). More than that, laboratory observations with Sytox green demonstrated that bacteria are often seen aggregating around the border cells in the aerial root mucilage (**Figure 7**), suggesting that these cells may play a role as extracellular traps as suggested for underground border cells (Driouich et al., 2013). Extracellular traps have been well described om the context of plant interactions with nematodes indeed but also bacteria (Hawes et al., 2011; Hawes et al., 2016).

We quantified mucilage volume and border cell quantity in the root tip released from underground roots and aerial roots in field experiments. Underground maize root tips produced ~20 µL of mucilage with ~59 border cells per µl, similarly to what has been published previously (Hawes et al., 1998). Maize accession L4 and L11 aerial roots produced 1-2 mL of mucilage per root and contained around 250-450 _˙_10^3^ border cells per mL of mucilage, while SM produced about 102 _˙_ 10^3^ border cells/mL in field conditions (**Supplementary Figure S7**). The present work is one of the few studies demonstrating the characteristics of aerial root border cells; however, more anatomical, and ecological studies are necessary to understand 1) the role these cells play in the mucilage secretion and 2) to establish a specific microenvironment for microbe population and efficient nitrogen fixation. The border cells described here could be a novel model for single-cell studies to understand the molecular mechanism driving biological nitrogen fixation in the mucilage secreted by aerial roots.

### Maize aerial roots secrete mucilage and respond to contact with water at the transcriptional level by regulating genes related to polysaccharide biosynthesis and degradation

We performed an RNA-seq experiment on whole aerial roots from SM maize and the B73. B73 develops a few thin aerial roots that produce only ~50 µL of mucilage after rain. We performed a time-course experiment with samples taken from both genotypes before exposure to water and then 2 and 24 hours after contact with water. As expected, genes implicated in water response were induced in both genotypes, and these genes include *Zm00001d010410* and *Zm00001d037779*, which encode aquaporins. Moreover, genes encoding proteins involved in polysaccharide biosynthesis were differentially expressed in the SM samples in response to water. Those genes encode specific glycosyltransferases and other transferases related to xyloglucan synthesis (**Figure 4**), suggesting that these genes could be involved in mucilage production and that this production occurs even before water exposure. We hypothesize that root cap cells and border cells synthesize mucilage polysaccharides before contact with water and that the polysaccharides are released post-contact. Border cells have been described to contain numerous storage granules (Newcomb, 1967; Feldman and Briggs, 1987). Some of these granules may contain “pre-made” mucilage polysaccharides. Also, we observed that genes encoding proteins involved in mucilage degradation were more induced in SM accession than in B73, suggesting that mucilage could be degraded into simple sugars by bacterial activity and by plant enzymes.

Furthermore, we observed that aerial roots express NH_4_^+^ and NO_3_^-^ transporter, putative peptide transporters, suggesting that NH_4_^+^ may not be the only form of nitrogen transferred from the microbial community of the plant. A study conducted by Dechorgnat et al. (2019) on the expression profile of ammonium and nitrate transporters in maize showed that, although expressed similarly in all organs, *ZmAMF1*.1 and *ZmAMF1*.2 presented different responses to nitrogen. In both roots and shoots, *ZmAMF1*.1 expression increased after nitrogen starvation and decreased after resupply (Dechorgnat et al., 2019). Here, *ZmAMF1*.1 increased after 24 hours in B73 and SM stage 1, indicating probable nitrogen starvation. However, *ZmAMF1*.1 decreased in SM stage 2 after 24 hours, suggesting nitrogen resupply. Considering that stage 2 roots have already gone through many cycles of watering and drying of the gel, and the water evaporates, it makes sense that the roots do not respond to starvation but are likely taking up ammonium at this stage. These results suggest that ammonium is one of the nitrogen currencies used by the aerial root of SM accessions.

The B73 maize genome contains two copies of the *NRT3*.1 gene: *ZmNRT3*.1A and *ZmNRT3*.1B. Neither has been characterized in maize (Plett et al., 2010). Here we demonstrated that NRT3.1B was expressed only in SM accessions. In the study of Dechorgnat et al. (2019), *ZmNRT3*.1B expression was detected in old leaves, and it was 100-fold less than its homolog *ZmNRT3*.1A. Again, here we show that level of expression of *ZmNRT3*.1B and *ZmNRT3*.1A are similar in the aerial root of SM accessions. Altogether this suggests a possible role for this uncharacterized gene to be specific to aerial roots.

Genes encoding glutamine synthetases (*Zm00001d017958* and *Zm00001d051804*) as well as glutamate dehydrogenase 2 (*Zm00001d016419)* are strongly induced after water treatments, consistent with prior observations that glutamine synthetase genes are up-regulated transcriptionally at uptake sites (Bernard and Habash, 2009). Additionally, a specific Hexosyltranferase (*Zm00001d020583*), overexpressed upon water treatment, has been significantly associated with traits related to low-nitrogen management conditions and is suggested to be a marker for assisted improvement in African tropical maize (Ertiro, 2018).

This study presented some limitations, especially regarding scarce literature on the specific topic, the plant size, and mucilage viscosity characteristics. However, altogether, the RNAseq experiments in maize aerial roots indicate a complex series of events, some of them triggered *via* water, involving different root development stages.

The mucilage environment likely represents a balanced combination of microbes that survive this environment and interact with the plant in a symbiotic manner. Several microbes and their metabolism could be better studied using synthetic communities (to reduce the microbial complexity) and systems biology (to model these interactions) toward understanding the basis of molecular interaction between plants and beneficial members at the maize aerial root environment to intentionally maintain the trait in plant breeding programs.

## Data availability statement

The RNAseq data presented in the study are deposited in the NCBI’'s Gene Expression Omnibus (Edgar et al., 2002) repository, accession number GSE168384 (https://www.ncbi.nlm.nih.gov/geo/query/acc.cgi?acc=GSE168384). The supplementary dataset present in this study can be found at in the Supplementary material https://www.frontiersin.org/articles/10.3389/fpls.2022.977056/full#supplementary-material.

## Author contributions

VP conceived the idea, analyzed the RNA-seq data, performed the border cells experiments, and wrote the manuscript. HH performed border cell experiments and wrote the manuscript. VI and CC performed the experiment in the 2019 summer season and the stats analysis. DJ performed the RNAseq experiment. P-MD, and SR performed the experiment and collected data from the 2014 summer season experiment. PZ conceived the idea and managed experiments at UC Davis along with AB. AB conceived the idea and managed experiments at UC Davis. J-MA conceived the idea, wrote the manuscript, raised funds, and managed the project. All authors contributed to the article and approved the submitted version.

## Acknowledgments

The authors of this publication would like to acknowledge the Community of Totontepec Villa De Morelos for providing the mucilage and genetic resources (*Zea mays*, primary breed OLOTON) used in the research reflected in his publication. We thank Claire Benezech and Juan P. Guttierez for underground border cell counting of Ames 19897 accession. We thank Shawn Kaeppler and Natalia de Leon Gatti for inbreed seeds donation; Junko Maeda and Bjorn Karlson for technical assistance; and the UC Davis sequencing facility for library construction assistance. This work was supported by grants from the US Department of Energy #DE-SC0021052 and the US Department of Agriculture grant #1024073 to J-MA.

## Conflict of interest

The authors declare that the research was conducted in the absence of any commercial or financial relationships that could be construed as a potential conflict of interest.

## Publisher’s note

All claims expressed in this article are solely those of the authors and do not necessarily represent those of their affiliated organizations, or those of the publisher, the editors and the reviewers. Any product that may be evaluated in this article, or claim that may be made by its manufacturer, is not guaranteed or endorsed by the publisher.

## Abbreviations

SM, Sierra Mixe; L1 to L14, Mexican accessions; TPM, transcripts per million; DEG, differentially expressed genes; T0, time zero, before adding water; T2, time two, two hours after adding water; T24, time twenty-four after adding water.

## References

[B1] Amicucci M. J. Galermo A. G. Guerrero A. Treves G. Nandita E. Kailemia M. J.. (2019). Strategy for structural elucidation of polysaccharides: Elucidation of a maize mucilage that harbors diazotrophic bacteria. Anal. Chem 91 (11), 7254–7265. doi: 10.1021/acs.analchem.9b00789 30983332

[B2] Andrews S. (2015). FASTQC a quality control tool for high throughput sequence data (Babraham Inst). Available at: https://www.bioinformatics.babraham.ac.uk/projects/fastqc/

[B3] Basu D. Wang W. Ma S. DeBrosse T. Poirier E. Emch K.. (2015). Two hydroxyproline galactosyltransferases, GALT5 and GALT2, function in arabinogalactan-protein glycosylation, growth and development in arabidopsis d. bassham, ed. PloS One 10 (5), e0125624. doi: 10.1371/journal.pone.0125624 25974423 PMC4431829

[B4] Bennett A. B. Pankievicz V. C. S. Ané J. M. (2020). A model for nitrogen fixation in cereal crops. Trends Plant Sci 25 (3), 226–235. doi: 10.1016/j.tplants.2019.12.004 31954615

[B5] Bernard S. M. Habash D. Z. (2009). The importance of cytosolic glutamine synthetase in nitrogen assimilation and recycling. New Phytol 182 (3), 608–620. doi: 10.1111/j.1469-8137.2009.02823.x 19422547

[B6] Bloch S. E. Clark R. Gottlieb S. S. Wood L. K. Shah N. Mak S.-M.. (2020). Biological nitrogen fixation in maize: Optimizing nitrogenase expression in a root-associated diazotroph. J. Exp. Bot 71 (15), 4591–4603. doi: 10.1093/jxb/eraa176 32267497 PMC7382387

[B7] Bray N. L. Pimentel H. Melsted P. Pachter L. (2016). Near-optimal probabilistic RNA-seq quantification. Nat. Biotechnol 34 (8), 888. doi: 10.1038/nbt0816-888d 27043002

[B8] Brigham L. A. Woo H. H. Hawes M. C. (1995). Root border cells as tools in plant cell studies. Methods Cell Biol 49, 377–387. doi: 10.1016/S0091-679X(08)61467-3 8531770

[B9] Brown L. K. George T. S. Neugebauer K. White P. J. (2017). The rhizosheath – a potential trait for future agricultural sustainability occurs in orders throughout the angiosperms. Plant Soil 419 (1–2), 115–128. doi: 10.1007/s11104-017-3220-2

[B10] Chiasson D. M. Loughlin P. C. Mazurkiewicz D. Mohammadidehcheshmeh M. Fedorova E. E. Okamoto M.. (2014). Soybean SAT1 (Symbiotic ammonium transporter 1) encodes a bHLH transcription factor involved in nodule growth and NH4+ transport. Proc. Natl. Acad. Sci. U. S. A. 111 (13), 4814–4819. doi: 10.1073/pnas.1312801111 24707045 PMC3977234

[B11] Clowes F. A. L. (1976). Cell production by root caps. New Phytol 77 (2), 399–407. doi: 10.1111/j.1469-8137.1976.tb01529.x

[B12] Curlango-Rivera G. Huskey D. A. Mostafa A. Kessler J. O. Xiong Z. Hawes M. C. (2013). Intraspecies variation in cotton border cell production: Rhizosphere microbiome implications. Am. J. Bot 100 (9), 1706–1712. doi: 10.3732/ajb.1200607 23942085

[B13] Dechorgnat J. Francis K. L. Dhugga K. S. Rafalski J. A. Tyerman S. D. Kaiser B. N. (2019). Tissue and nitrogen-linked expression profiles of ammonium and nitrate transporters in maize. BMC Plant Biol 19 (1), 206. doi: 10.1186/s12870-019-1768-0 31109290 PMC6528335

[B14] Dechorgnat J. Nguyen C. T. Armengaud P. Jossier M. Diatloff E. Filleur S.. (2011). From the soil to the seeds: The long journey of nitrate in plants. J. Exp. Bot 62 (4), 1349–1359. doi: 10.1093/jxb/erq409 21193579

[B15] Driouich A. Follet-Gueye M.-L. Vicré-Gibouin M. Hawes M. (2013). Root border cells and secretions as critical elements in plant host defense. Curr. Opin. Plant Biol 16 (4), 489–495. doi: 10.1016/j.pbi.2013.06.010 23856080

[B16] Driouich A. Smith C. Ropitaux M. Chambard M. Boulogne I. Bernard S.. (2019). Root extracellular traps versus neutrophil extracellular traps in host defence, a case of functional convergence? Biol. Rev. Cam. Phil. Soc 94 (5), 1685–1700. doi: 10.1111/brv.12522 31134732

[B17] Duff R. B. (1965). The occurrence of apiose in lemna (duckweed) and other angiosperms. Biochem. J. 94, 768–772. doi: 10.1042/bj0940768 14340070 PMC1206615

[B18] Edgar R. Domrachev M. Lash AE. (2002). Gene Expression Omnibus: NCBI gene expression and hybridization array data repository. Nucleic Acids Res 30 (1), 207–10. doi: 10.1093/nar/30.1.207 PMC99122 11752295

[B19] Ellis M. Egelund J. Schultz C. J. Bacic A. (2010). Arabinogalactan-proteins: Key regulators at the cell surface? Plant Physiol 153 (2), 403–419. doi: 10.1104/pp.110.156000 20388666 PMC2879789

[B20] Eom J. S. Chen L. Q. Sosso D. Julius B. T. Lin I. W. Qu X. Q.. (2015). SWEETs, transporters for intracellular and intercellular sugar translocation. Curr. Opin. Plant Biol 25, 53–62. doi: 10.1016/j.pbi.2015.04.005 25988582

[B21] Ertiro B. T. (2018). Prospects for marker assisted improvement of African tropical maize germplasm for low nitrogen tolerance. (University of the Free State). Available at: https://scholar.ufs.ac.za/handle/11660/9093

[B22] Feldman L. J. Briggs W. R. (1987). Light-regulated gravitropism in seedling roots of maize. Plant Physiol 83, 241–243. doi: 10.1104/pp.83.2.241 11539030 PMC1056339

[B23] Galloway A. F. Akhtar J. Marcus S. E. Fletcher N. Field K. Knox P. (2020). Cereal root exudates contain highly structurally complex polysaccharides with soil-binding properties. Plant J Cell Mol. Biol. 103 (5), 1666–1678. doi: 10.1111/tpj.14852 32463959

[B24] Guinel F. C. McCully M. E. (1986). Some water-related physical properties of maize root-cap mucilage. Plant. Cell Environ. 9 (8), 657–666. doi: 10.1111/j.1365-3040.1986.tb01624.x

[B25] Hawes M. Allen C. Turgeon B. G. Curlango-Rivera G. Minh Tran T. Huskey D. A. (2016). Root border cells and their role in plant defense. Annu. Rev. Phytopathol. 54, 143–161. doi: 10.1146/annurev-phyto-080615-100140 27215971

[B26] Hawes M. C. Brigham L. A. Wen F. Woo H. H. Zhu Y. (1998). Function of root border cells in plant health: Pioneersin the rhizosphere. Annu. Rev. Phytopathol 36 (1), 311–327. doi: 10.1146/annurev.phyto.36.1.311 15012503

[B27] Hawes M. C. Curlango-Rivera G. Wen F. White G. J. VanEtten H. D. Xiong Z. (2011). Extracellular DNA: The tip of root defenses? Plant Sci Int. J. Exp. Plant Biol. 180 (6), 741–745. doi: 10.1016/j.plantsci.2011.02.007 21497709

[B28] Hawes M. C. Gunawardena U. Miyasaka S. Zhao X. (2000). The role of root border cells in plant defense. Trends Plant Sci 5 (3), 128–133. doi: 10.1016/S1360-1385(00)01556-9 10707079

[B29] Hawes M. C. Pueppke S. G. (1986). Sloughed peripheral root cap cells: yield from different species and callus formation from single cells. Am. J. Bot 73 (10), 1466–1473. doi: 10.1002/j.1537-2197.1986.tb10892.x

[B30] Iijima M. Griffiths B. Bengough A. G. (2000). Sloughing of cap cells and carbon exudation from maize seedling roots in compacted sand. New Phytol 145 (3), 477–482. doi: 10.1046/j.1469-8137.2000.00595.x 33862902

[B31] Knox O. G. G. Curlango-Rivera G. Huskey D. A. Hawes M. C. (2020). Border cell counts of Bollgard3 cotton and extracellular DNA expression levels. Euphytica Nether. J Plant Breeding 216 (9), 142. doi: 10.1007/s10681-020-02678-8

[B32] Koegel S. Ait Lahmidi N. Arnould C. Chatagnier O. Walder F. Ineichen K.. (2013). The family of ammonium transporters (AMT) in *Sorghum bicolor*: Two AMT members are induced locally, but not systemically in roots colonized by arbuscular mycorrhizal fungi. New Phytol 198 (3), 853–865. doi: 10.1111/nph.12199 23461653

[B33] Kumar N. Iyer-Pascuzzi A. (2020). Shedding the last layer: Mechanisms of root cap cell release. Plants 9 (3). doi: 10.3390/plants9030308 PMC7154840 32121604

[B34] Ma W. Muthreich N. Liao C. Franz-Wachtel M. Schütz W. Zhang F.. (2010). The mucilage proteome of maize (*Zea mays* l.) primary roots. J. Proteome Res 9 (6), 2968–2976. doi: 10.1021/pr901168v 20408568

[B35] Newcomb E. H. (1967). Tubule-bearing vesicles associated with slime-body formation in differentiating cells of bean root tips. Science 158 (3800), 532–3. doi: 10.1126/science.158.3800.532-c 17749104

[B36] Pankievicz V. C. S. Irving T. B. Maia L. G. S. Ané J. M. (2019). Are we there yet? The long walk towards the development of efficient symbiotic associations between nitrogen-fixing bacteria and non-leguminous crops. BMC Biol 17 (1), 99. doi: 10.1186/s12915-019-0710-0 31796086 PMC6889567

[B37] Pimentel H. Bray N. L. Puente S. Melsted P. Pachter L. (2017). Differential analysis of RNA-seq incorporating quantification uncertainty. Nat. Methods 14 (7), 687–690. doi: 10.1038/nmeth.4324 28581496

[B38] Plett D. Toubia J. Garnett T. Tester M. Kaiser B. N. Baumann U. (2010). Dichotomy in the NRT gene families of dicots and grass species. PloS One 5 (12), e15289. doi: 10.1371/journal.pone.0015289 21151904 PMC2997785

[B39] Raudvere U. Kolberg L. Kuzmin I. Arak T. Adler P. Peterson H.. (2019). G:Profiler: A web server for functional enrichment analysis and conversions of gene lists, (2019 Update). Nucleic Acids Res 47 (W1), W191–W198. doi: 10.1093/nar/gkz369 31066453 PMC6602461

[B40] Rovira A. D. (1969). Plant root exudates. Bot. Rev 35 (1), 35–57. doi: 10.1007/BF02859887

[B41] Schindelin J. Arganda-Carreras I. Frise E. Kaynig V. Longair M. Pietzsch T.. (2012). Fiji: An open-source platform for biological-image analysis. Nat. Methods 9 (7), 676–682. doi: 10.1038/nmeth.2019 22743772 PMC3855844

[B42] Seifert G. J. Roberts K. (2007). The biology of arabinogalactan proteins. Annu. Rev. Plant Biol 58, 137–161. doi: 10.1146/annurev.arplant.58.032806.103801 17201686

[B43] Steffens B. Rasmussen A. (2016). The physiology of adventitious roots. Plant Physiol 170 (2), 603–617. doi: 10.1104/pp.15.01360 26697895 PMC4734560

[B44] Tran T. M. MacIntyre A. Hawes M. Allen C. (2016). Escaping underground nets: Extracellular DNases degrade plant extracellular traps and contribute to virulence of the plant pathogenic bacterium *Ralstonia solanacearum* S.Y. he, ed. PloS Pathog 12 (6), e1005686. doi: 10.1371/journal.ppat.1005686 27336156 PMC4919084

[B45] Van Deynze A. Zamora P. Delaux P. M. Heitmann C. Jayaraman D. Rajasekar S.. (2018). Nitrogen fixation in a accession of maize is supported by a mucilage-associated diazotrophic microbiota. PloS Biol 16 (8), e2006352. doi: 10.1371/journal.pbio.2006352 30086128 PMC6080747

[B46] Wang P. Chen X. Goldbeck C. Chung E. Kang B.-H. (2017). A distinct class of vesicles derived from thetrans-Golgi mediates secretion of xylogalacturonan in the root border cell. The Plant Journal: For Cell and Molecular Biology 92 (4), 596–610. doi: 10.1111/tpj.13704 28865155

[B47] Yan Z. Bo C. Shibin G. Tingzhao R. (2014). Biological characters of root border cells development in maize (*Zea mays*). Biotechnology. doi: 10.3923/biotech.2014.89.98

