# Supplementary figures and images for: Nitrogen fixation and mucilage production on maize aerial roots is controlled by aerial root development and border cell functions
Source: Front Plant Sci. 2022 Oct 6;13:977056. doi: 10.3389/fpls.2022.977056 (PMC9583020; doi:10.3389/fpls.2022.977056)

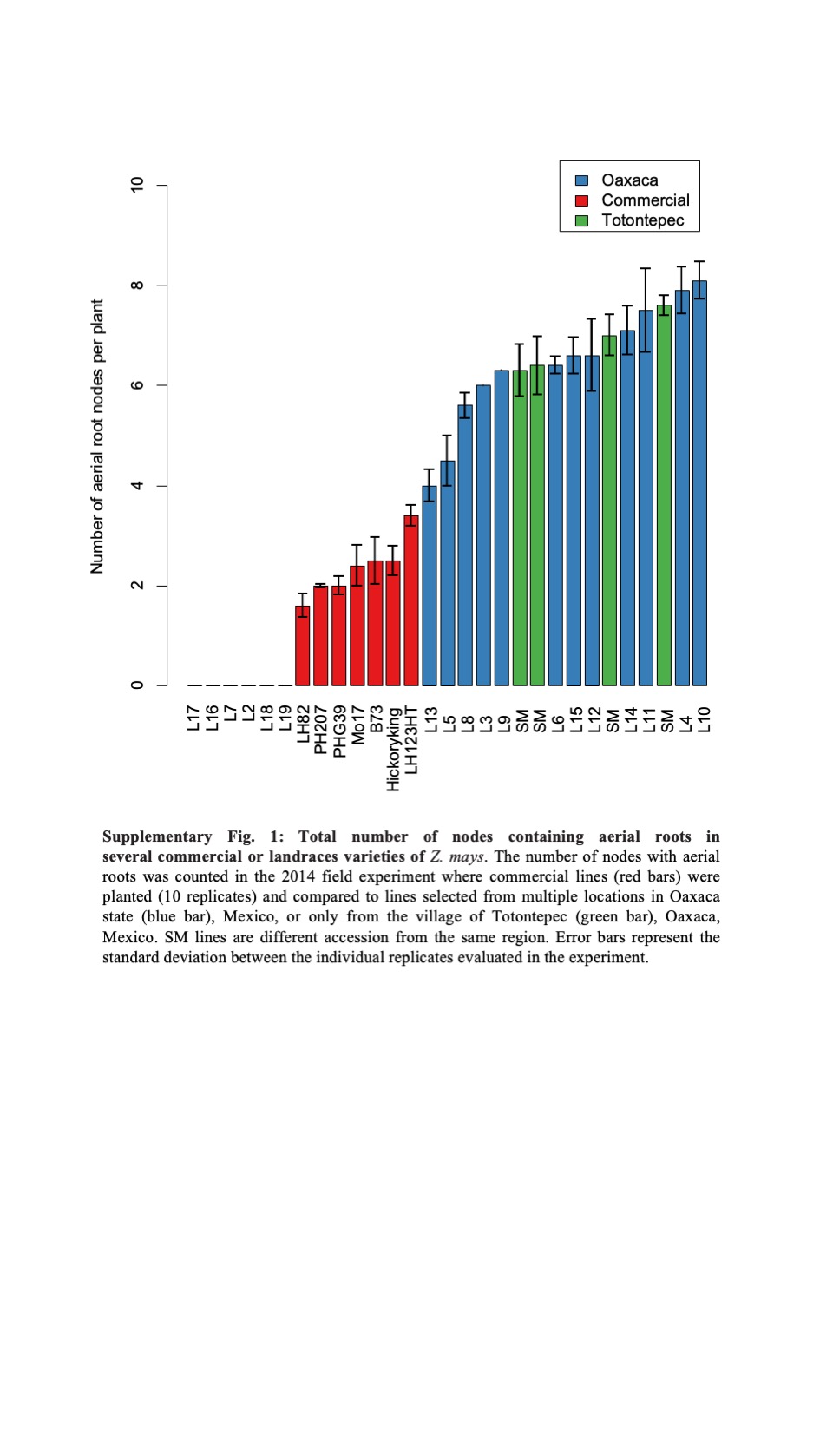

Supplement: Supplementary file 1 [file Image_1.jpeg]

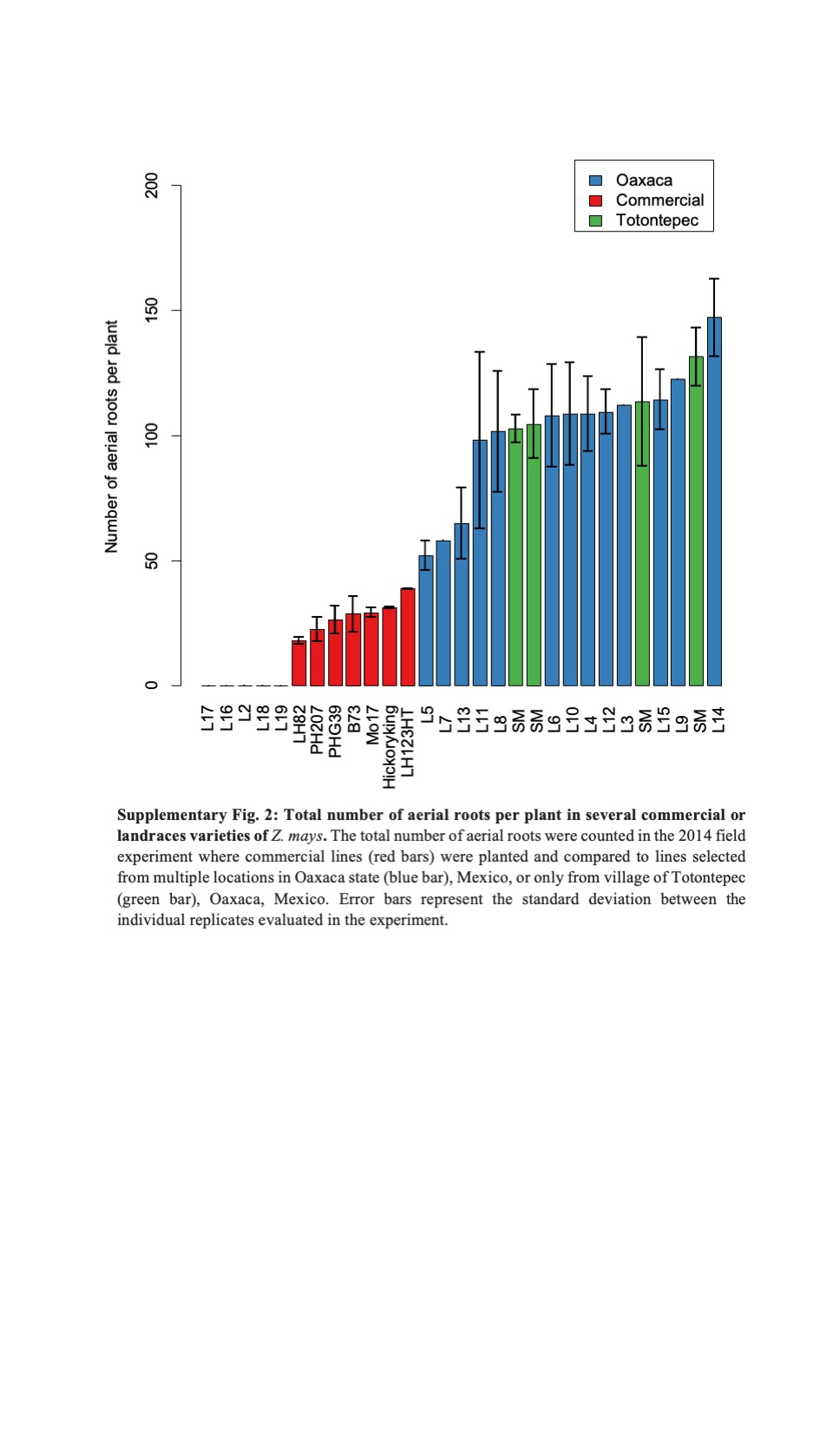

Supplement: Supplementary file 2 [file Image_2.jpeg]

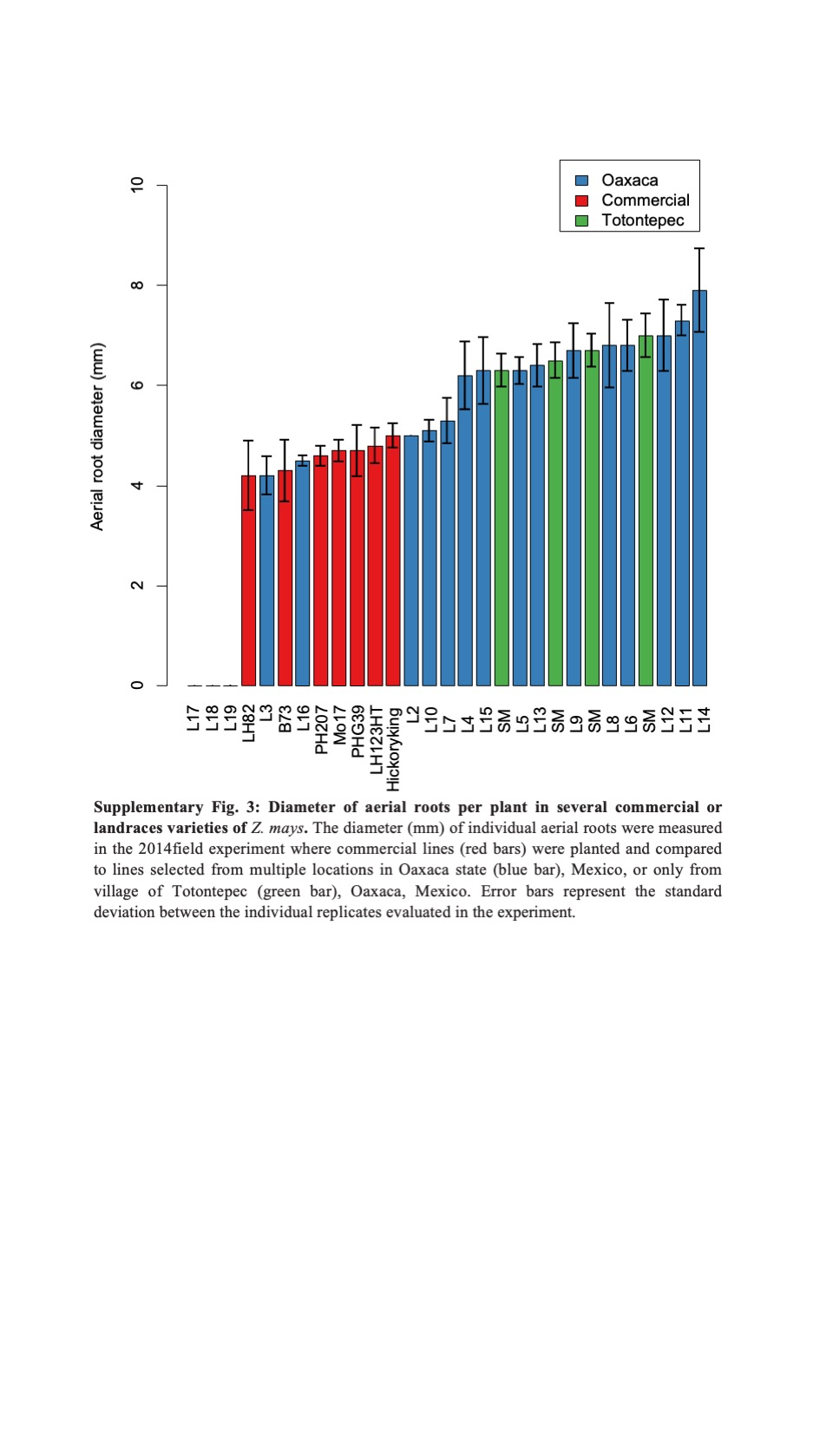

Supplement: Supplementary file 3 [file Image_3.jpeg]

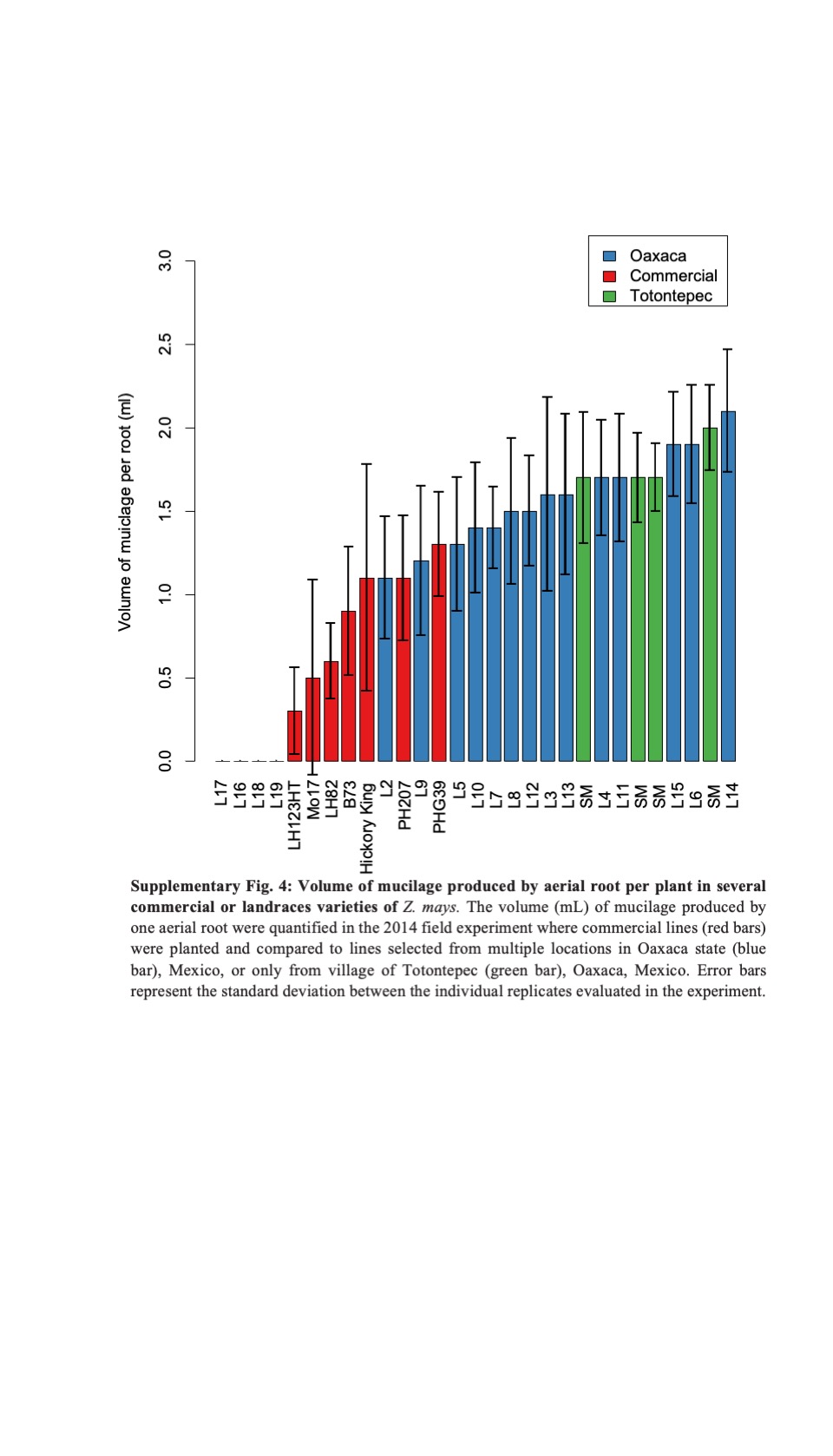

Supplement: Supplementary file 4 [file Image_4.jpeg]

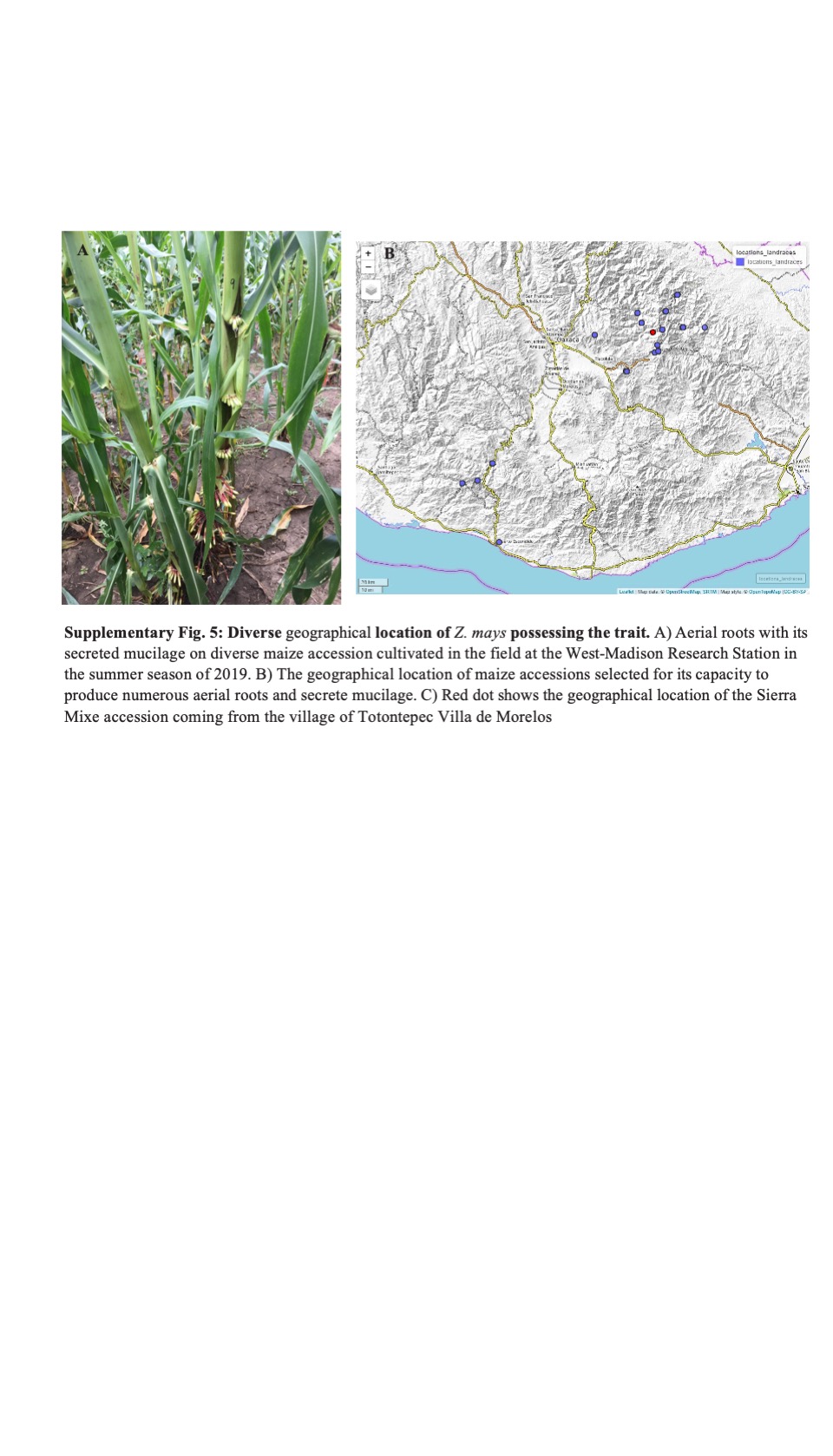

Supplement: Supplementary file 5 [file Image_5.jpeg]

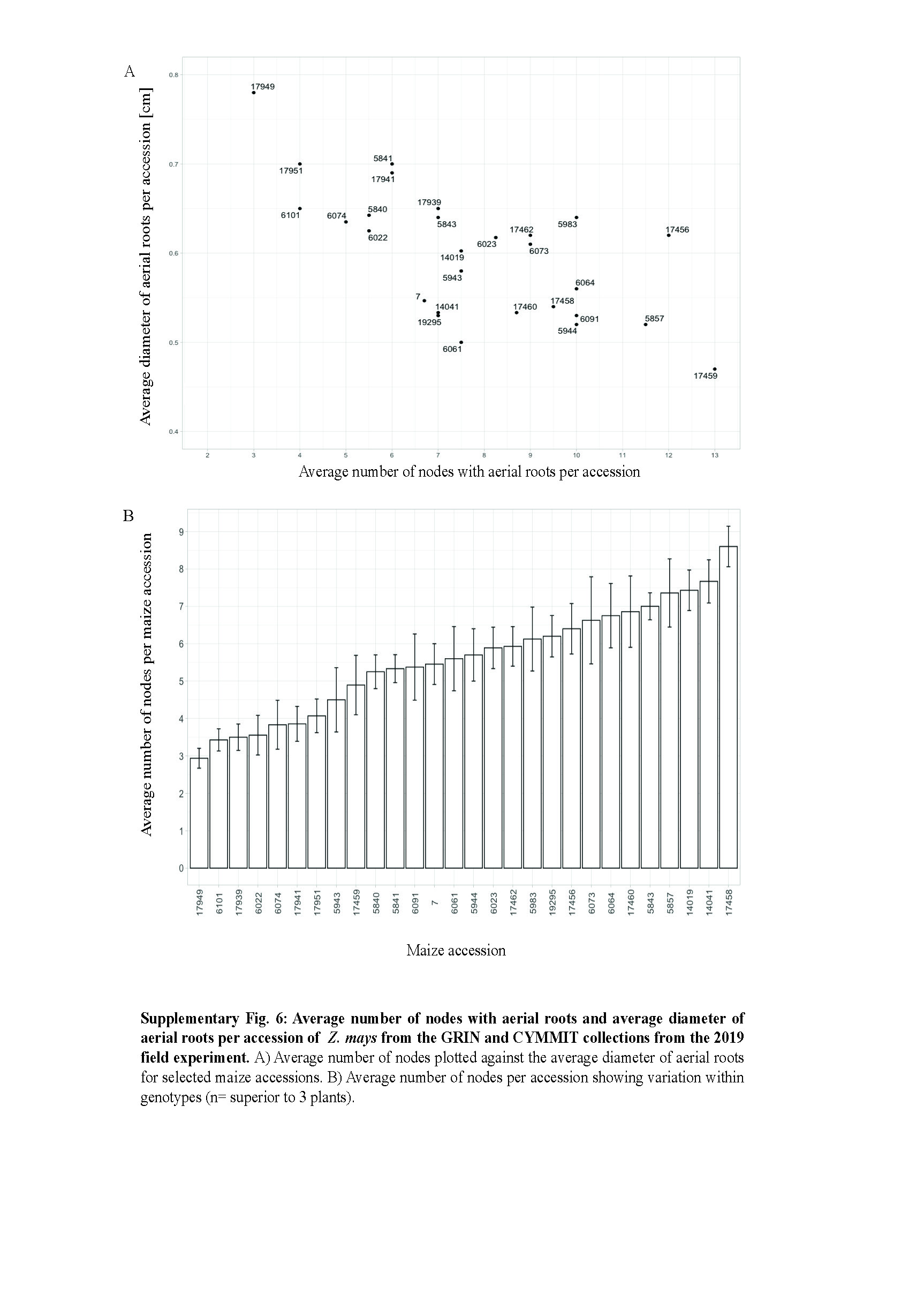

Supplement: Supplementary file 6 [file Image_6.jpeg]

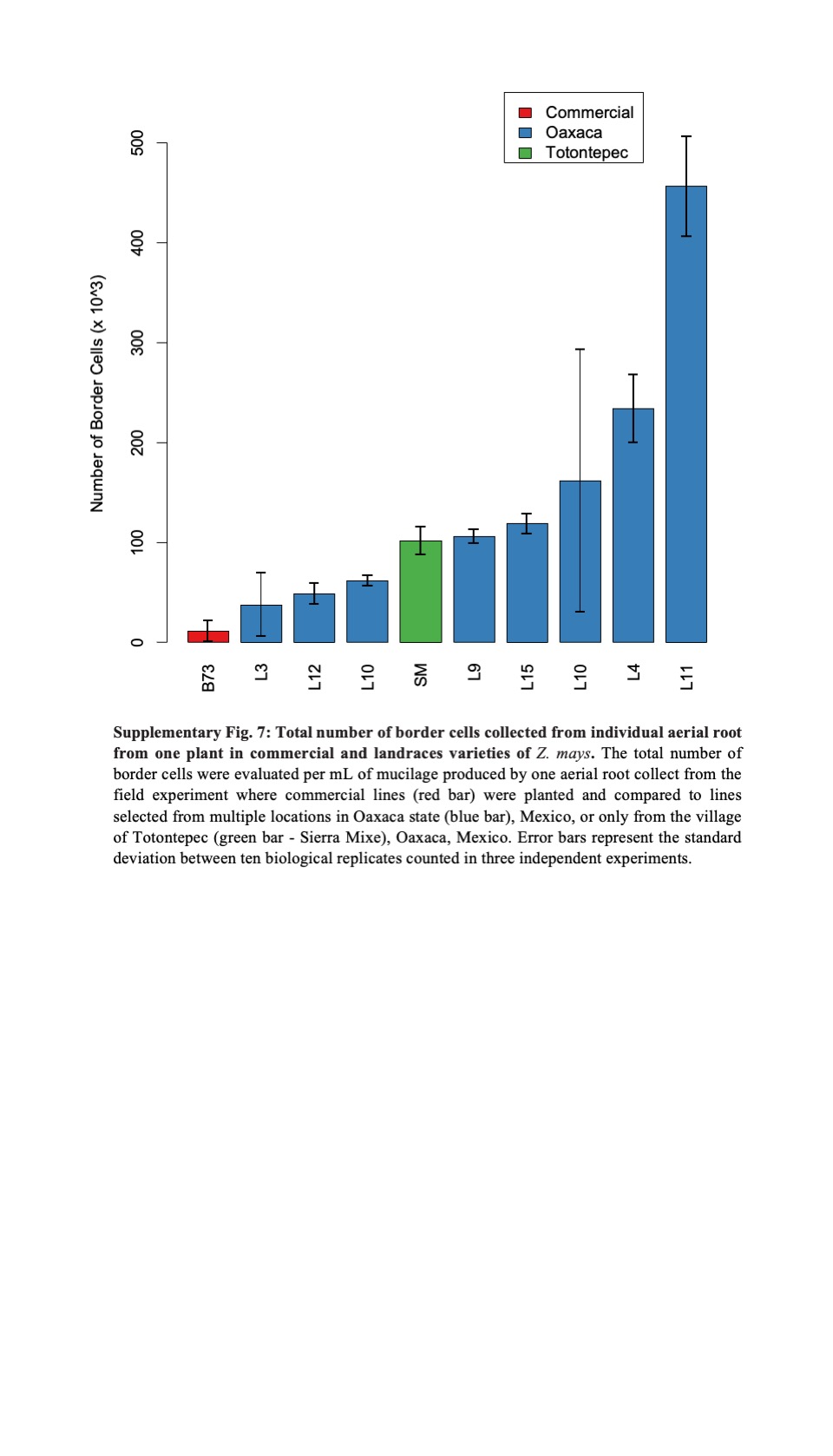

Supplement: Supplementary file 7 [file Image_7.jpeg]
